# Wolf outside, dog inside? The genomic make-up of the Czechoslovakian Wolfdog

**DOI:** 10.1186/s12864-018-4916-2

**Published:** 2018-07-13

**Authors:** Romolo Caniglia, Elena Fabbri, Pavel Hulva, Barbora Černá Bolfíková, Milena Jindřichová, Astrid Vik Stronen, Ihor Dykyy, Alessio Camatta, Paolo Carnier, Ettore Randi, Marco Galaverni

**Affiliations:** 1 Area per la Genetica della Conservazione, ISPRA, Ozzano dell’Emilia, Bologna, Italy; 2 Department of Zoology, Charles University in Prague, Prague, Czech Republic; 3 Department of Biology and Ecology, Ostrava University, Ostrava, Czech Republic; 4 Faculty of Tropical AgriSciences, Czech University of Life Sciences Prague, Prague, Czech Republic; 5 Department of Chemistry and Bioscience, Aalborg University, Aalborg Øst, Denmark; 6 Department of Zoology, Ivan Franko National University of Lviv, Lviv, Ukraine; 7 CLC-Italia, Conegliano, Treviso Italy; 8 Department of Comparative Biomedicine and Food Science, University of Padova, Padova, Italy; 9 Dipartimento BIGEA, Università di Bologna, Bologna, Italy; 10 Department 18/ Section of Environmental Engineering, Aalborg University, Aalborg Øst, Denmark; 11 Area Conservazione, WWF Italia, Rome, Italy

**Keywords:** Admixture history, Czechoslovakian Wolfdog, Demographic history, Genome ancestry, Genome-wide differentiation, Hybridization, Selection

## Abstract

**Background:**

Genomic methods can provide extraordinary tools to explore the genetic background of wild species and domestic breeds, optimize breeding practices, monitor and limit the spread of recessive diseases, and discourage illegal crossings. In this study we analysed a panel of 170k Single Nucleotide Polymorphisms with a combination of multivariate, Bayesian and outlier gene approaches to examine the genome-wide diversity and inbreeding levels in a recent wolf *x* dog cross-breed, the Czechoslovakian Wolfdog, which is becoming increasingly popular across Europe.

**Results:**

Pairwise *F*_ST_ values, multivariate and assignment procedures indicated that the Czechoslovakian Wolfdog was significantly differentiated from all the other analysed breeds and also well-distinguished from both parental populations (Carpathian wolves and German Shepherds). Coherently with the low number of founders involved in the breed selection, the individual inbreeding levels calculated from homozygosity regions were relatively high and comparable with those derived from the pedigree data. In contrast, the coefficient of relatedness between individuals estimated from the pedigrees often underestimated the identity-by-descent scores determined using genetic profiles. The timing of the admixture and the effective population size trends estimated from the LD patterns reflected the documented history of the breed. Ancestry reconstruction methods identified more than 300 genes with excess of wolf ancestry compared to random expectations, mainly related to key morphological features, and more than 2000 genes with excess of dog ancestry, playing important roles in lipid metabolism, in the regulation of circadian rhythms, in learning and memory processes, and in sociability, such as the *COMT* gene, which has been described as a candidate gene for the latter trait in dogs.

**Conclusions:**

In this study we successfully applied genome-wide procedures to reconstruct the history of the Czechoslovakian Wolfdog, assess individual wolf ancestry proportions and, thanks to the availability of a well-annotated reference genome, identify possible candidate genes for wolf-like and dog-like phenotypic traits typical of this breed, including commonly inherited disorders. Moreover, through the identification of ancestry-informative markers, these genomic approaches could provide tools for forensic applications to unmask illegal crossings with wolves and uncontrolled trades of recent and undeclared wolfdog hybrids.

**Electronic supplementary material:**

The online version of this article (10.1186/s12864-018-4916-2) contains supplementary material, which is available to authorized users.

## Background

Since the late Pleistocene, humans have indirectly or actively pursued the domestication of wild animal and plant species for food and material production, safety and entertainment purposes [1]. Over time, a growing number of species was selected through controlled crossings in order to artificially fix or enhance the desired productive, aesthetic or behavioural traits, resulting in varieties and breeds more useful for human benefits but progressively more differentiated from their wild progenitors [2]. However, an opposite trend is currently developing in order to obtain more balanced varieties in terms of nutritive components or individuals with traits more similar to their ancestors, partially reverting the effects of domestication [1]. A prominent example of such a tendency is represented by the growing popularity of commercialized wolfdog breeds, such as the Saarloos Wolfdog, the Lupo Italiano, the Kunming Wolfdog, the American Wolfdog and the Czechoslovakian Wolfdog, which were created by the deliberate crossing of wolf-like or ancient breeds (e.g. the German Shepherd, the Siberian Husky and the Alaskan Malamute) with wild wolves [3], representing extreme cases of anthropogenic hybridization [4]. The Czechoslovakian Wolfdog (CWD) is the most widespread among such breeds, currently accounting 24,982 registered individuals worldwide (CLC-Italia database, http://clc-italia.it). CWDs are the result of a military experiment carried out in Czechoslovakia during the 1950s. The aim was to create a new breed showing the temperament and controllability of the German Shepherd together with the strength and sensorial abilities of the Carpathian wolf to assist the Czechoslovakian military to patrol the country’s borders. The first litter was obtained in 1958 by crossing a female Carpathian wolf (Brita) and a male German Shepherd (Cézar). The progeny was crossbred afterwards, with only four additional crossings with wolves in 1960 (again with the female wolf Brita), 1968 (male Carpathian wolf Argo), 1974 (male Carpathian wolf Šarik) and 1983 (female Carpathian wolf Lejdy). At the end of the military experiment, after a temporary recognition in 1989, in 1999 the breed was officially recognized with its own standard by the Fédération Cynologique Internationale (FCI), which requires a wolf-like morphology but also tameness and loyalty towards the master (FCI Standard N° 332). Afterwards, any crossing with wolves or other dog breeds was strictly forbidden and the animal phenotypes now appear to be steadily consistent with the breed standards (FCI Standard N° 332).

However, a series of problems can arise from such a peculiar history since a recent breed that originated from a very limited number of founders could be expected to carry reduced genetic variability and high levels of inbreeding, although such a threat was not documented by the results from preliminary genetic studies performed with a restricted number of genetic loci including autosomal microsatellites, Y-chromosome and mitochondrial DNA markers [3, 5–7]. Second, several recessive diseases or disorders, frequently found in German Shepherds, can also affect CWDs in cases of high homozygosity, such as hip dysplasia, a multifactorial disease affecting the femoral joint, which has been observed in 14.69% of the CWD individuals, with a heritability of 28.9% (P. Carnier, personal communication, calculated with the U-WGI software based on the CLC-Italia database, http://clc-italia.it). However, a number of other diseases have been recently described in CWDs. Some of them have a known genetic basis, such as pituitary dwarfism (originated by a single mutation on the *LHX3* gene; [8]) and degenerative myelopathy (mainly caused by a recessive mutation on the *SOD1* gene; [9]), whereas others have still unknown or multifactorial bases, such as haemangiosarcoma, cryptorchidism, sub-aortic stenosis and endocrine pancreatic insufficiency (a review can be found in [10]. A third controversy is represented by illegal crossings with wolves aiming to produce animals with a more wolf-like appearance to be sold at a higher price than standard CWDs (A. Camatta, personal communication). However, handling those “parlour wolves” might be far from simple due to their less predictable temperament likely caused by the disruption of the genetic composition and epistatic interactions established during several decades of artificial selection of behavioural traits in CWDs, as morphological and behavioural traits in canids can be tightly linked [11–14]. Moreover, if such animals are abandoned or escape into the wild, given their higher similarity, they could more easily hybridize with wolves than other breeds, contributing to the introgression of dog alleles into the wolf genome, which represents a serious conservation concern for several wolf populations [3, 6, 15, 16].

Nowadays, genomic tools provide unprecedented opportunities to explore the genome-wide genetic background of a breed, increase the efficiency of selective breeding practices, monitor and limit the spread of recessive diseases, and discourage illegal crossings [17–19]. However, such possibilities have not been exploited yet in the case of wolfdogs and only a few studies have so far investigated the genetic composition of CWDs [3, 5–7].

Therefore, by applying a 170k canine SNP chip and a combination of multivariate, Bayesian and gene search approaches, in this study we aim to: 1) compare the genome-wide diversity of CWDs and their differentiation from parental populations (Carpathian wolves and German Shepherds) and from other common breeds [17, 20]; 2) compare genetic diversity and demographic parameters assessed from genome-wide markers to those inferred from registered pedigrees; 3) reconstruct the ancestry of wolf-derived and dog-derived chromosomal blocks and, 4) thanks to the availability of the annotated dog reference genome, identify candidate genes that could codify for phenotypical traits typical of the breed.

## Methods

### Sample collection, DNA extraction and SNP genotyping

Using a DNeasy Tissue Kit (Qiagen Inc., Hilden, Germany) and following the manufacturer’s instructions, we extracted DNA from blood samples of 12 unrelated CWDs and from muscular tissue samples of 12 unrelated Carpathian wolves. No animal was sacrificed for the purposes of this study. CWD blood samples were collected from 2003 to 2013 in the Czech Republic by veterinaries, from animals in healthy conditions, with the permission and assistance of the owners, minimizing any possible stress. The dog owners also authorised the genetic data obtained from their animals to be used in this study, while maintaining their identity confidential. However, two owners did not gave their permission to use the pedigree data associated to their dogs, therefore the individual pedigree-based analyses were based upon the 10 remaining CWDs. Wolf tissue samples were collected from eight Western Ukrainian, three Slovakian and one Polish wolves [21], randomly sampled from different packs in order to avoid inbreeding or sampling bias and to be as much as possible representative of the Carpathian population. Tissues were collected, for purposes other than this project, from animals found dead or legally harvested by hunters with special permission under legal hunting quota limits. No ethics permit was required since wolf sample collection involved only dead animals. All samples were collected by specialized technician personnel.

CWD and Carpathian wolf DNA samples were genotyped at *c.* 170k SNPs using the CanineHD BeadChip microarray (Illumina, Inc., San Diego, California, USA), following the Infinium HD Ultra Assay protocol and calling genotypes with GenomeStudio (http://www.illumina.com/documents/products/datasheets/datasheet_genomestudio_software.pdf).

For comparative purposes, we then added publicly available genotypes from 355 dogs belonging to 30 breeds that were genotyped with the same 170k SNP microarray in the LUPA project, realized for the genetic mapping of a number of canine diseases [17, 20]. In particular, this dataset included also 12 German Shepherds that, thanks to their limited within-breed variation [20] and stable breeding practices, can represent a very good proxy of the original dog founders of the Czechoslovakian Wolfdog breed.

### Data filtering

The genotypes from these 379 individuals were filtered in the SNP&Variant Suite 8.0.1 (SVS, Golden Helix Inc., Bozeman, MT) discarding samples and SNPs with call rates ≤ 95% and all loci mapping on chromosomes X and Y (quality-pruned dataset). Genotypes were further filtered to discard loci in linkage disequilibrium (LD) by Plink 1.07 [22], using the *--dog* option in order to manage the correct number of chromosomes and removing SNPs with pairwise genotypic associations r^2^ > 0.2 calculated along sliding windows of 50 SNPs (LD-pruned dataset).

### Summary statistics, assignment and admixture tests

A pairwise *F*_ST_ matrix of genetic distance [23] among groups, values of observed heterozygosity (*H*_o_) and the inbreeding coefficient (*F*) within groups were estimated from the quality-pruned dataset in SVS. To visualize the distribution of genotypes in the genetic space, an exploratory principal component analysis (PCA; [24]) was performed in SVS using the quality-pruned dataset and the additive genetic model [25].

We then ran assignment tests in Admixture 1.23 [26] on the LD-pruned dataset of CWDs, Carpathian wolves and German Shepherds, assuming *K* values from 1 to 5, to assign each sample to its population of origin and to evaluate the level of admixture in CWDs. The most likely number of clusters was identified based on the lowest cross-validation error [26] and results were plotted in R 3.0.2 (www.r-project.org).

A more accurate reconstruction of the parental proportions of ancestry in CWDs was achieved by the PCA-based admixture deconvolution approach implemented in PCAdmix 1.0 [27, 28], which was run with blocks of 10 consecutive, non-overlapping SNPs. For each CWD, we calculated the average genome-wide proportion of blocks assigned to each reference population. We then compared it to the percentage of wolf ancestry estimated from the CWD pedigrees with the software BreedMate Pedigree Explorer (www.breedmate.com).

### Runs of homozygosity, linkage and relatedness

The quality-pruned dataset was also used in SVS to assess the mean number and the mean length of runs of homozygosity (ROH) within groups to provide estimates of the inbreeding levels due to autozygosity, expecting proportionally longer ROHs in more recently inbred populations, given that recombination had less time to reduce their length [29, 30]. We then compared the distribution of ROHs for each individual CWD, Carpathian wolf and German Shepherd, and estimated their frequency of ROHs (F_ROH_), calculated as the proportion of ROHs on the genome length spanned by the analysed SNPs, which are a better proxy of the inbreeding levels of an individual since F_ROH_ are less prone than *F* statistics to underestimate inbreeding in populations with recently reduced effective sizes [31–33].

Values of F_ROH_ in CWDs were then compared to the values of inbreeding estimated from their pedigrees as coefficient of inbreeding (COI) with the software U-WGI in order to evaluate the concordance of such approaches in quantifying inbreeding. Similarly, we assessed the levels of relatedness by computing the pairwise identity-by-descent (IBD) scores between individuals in CWDs, Carpathian wolves and German Shepherds using SVS, representing genome-wide levels of relatedness. IBD values found in CWDs were then compared to the coefficient of relatedness (COR) computed from pedigree data (CLC-Italia database, http://clc-italia.it) with the software BreedMate Pedigree Explore.

We also assessed the LD patterns by estimating the physical distance at which the r^2^ coefficient decayed below a threshold of 0.1.

### Demographic trends and admixture time in Czechoslovakian Wolfdogs

We reconstructed the trends in the Czechoslovakian Wolfdog effective population size (*N*_*E*_) using the equation E(r^2^) = [1/(1+4 *N*_*E*_ *c*) + 1/n], where r^2^ is the squared correlation of genotypic association between autosomal SNPs (representing the extent of LD), *c* is the genetic distance between SNPs in Morgans (assuming 100 Mb = 1 Morgan) and 1/n is the correction factor for small sample sizes[34, 35]. In this way we estimated demographic changes that occurred 1 to 20 generations ago that, considering a dog generation time of 3 years [36], correspond to 3–60 years in the past and thus include the whole history of the breed. We expected *N*_*E*_ to increase at every crossing with additional wolves, then to decrease steadily, since only a portion of the individuals were used for breeding and the time since the breed formation, given the current population size, is not significant in accumulating new variants (*p* = mu × n. gen. × *N*_*E*_ = 1 × 10^-8^ × 20 × 20,000 = 0.004).

We reconstructed chromosomal haplotypes for CWDs, Carpathian wolves and German Shepherds in Shapeit 2.837 [37] using the quality-pruned dataset, standard parameters and dog recombination maps derived from [38], referred to the canFam2 dog genome assembly (namely the same build the SNP array was designed on). We then estimated the average timing of the admixture events between the parental populations of CWDs using Alder 1.03 [39], which exploits information derived from the haplotype structure and the extent of LD decay among neighbouring loci, assuming a generation time of 3 years [36]. Moreover, we assessed the number of generations since the admixture for each individual also using the number of switches from German Shepherd to Carpathian wolf ancestry blocks (or *vice versa*) and the formula developed by [40], modified according to the dog genome length, conditional on the proportion of admixture estimated from PCAdmix. Summary plots across all samples were then compared with those obtained from Alder, with the demographic trajectories estimated from LD and with the known history of the breed.

### Estimating wolf and dog local genome ancestry

Regions with an excess of wolf or dog contributions were first identified based on PCAdmix results, searching for chromosomal regions where all the analysed CWDs presented only wolf-like or only dog-like haplotypes (corresponding to 100% wolf or dog ancestry 100%, respectively).

Second, we identified genomic regions that in all CWDs were included within a ROH, likely indicating strong selective pressures acting on the genomic surroundings.

Third, we identified the SNPs most differentiating German Shepherds from Carpathian wolves (*F*_ST(GSh-WCA)_ = 1, calculated in SVS), indicative of sharp genetic differences between the two groups. Among them, we retained the 1% with the lowest *F*_ST_ differentiation between CWDs and Carpathian wolves (‘wolf-like SNPs’) and between CWDs and German Shepherds (‘dog-like SNPs’), which correspond to genomic positions where CWDs present a strong similarity to only one of the two parentals. The same reasoning was applied to blocks of 10 consecutive SNPs, which should identify positions where differentiation involves chromosomal segments instead of single SNPs.

After removing all sites with any missing data from the LD pruned dataset, a fourth set of outliers was selected by exploiting the ability of the software BGC (Bayesian Genomic Cline analysis; [41]) to identify SNPs with an excess of ancestry in one of the two parental populations compared to random expectations [41]. Specifically, we retained as outliers the SNPs falling in the 1^st^ lower or upper percentile of the alpha parameter distribution and whose confidence intervals (CI) did not include the value 0, indicating an excess of either wolf or dog alleles.

The fifth and last panel of outliers was composed of all the SNPs identified as significant at *p* < 0.05 by BayeScan [42], a software that detects loci whose allele frequency differs between two populations significantly more than their average genome-wide distance, comparing CWDs *vs.* German Shepherds and CWDs *vs.* Carpathian wolves.

### Gene search and gene ontology

Subsequently we selected the genomic intervals surrounding each outlier SNP or block by including 50 Kb on each side [20, 43]. We then translated their coordinates from canFam2 to canFam3 reference assembly using the *liftover* tool in the UCSC Genome Browser (https://genome.ucsc.edu/cgi-bin/hgLiftOver) and retrieved the genes included in each genomic interval from the in Ensembl gene annotation 87 in Biomart (http://www.ensembl.org/biomart/martview/).

The two lists of genes obtained (wolf-like genes, dog-like genes) were then analysed for their possible enrichment towards any category included in the Gene Ontology (GO) - Biological Processes (BP) and in the Human Phenotypes (HP) ontology databases. Enrichment was tested in *gProfiler* [44], only retaining categories having a size domain of at most 500 terms and being significant after Benjamini-Hochberg correction for multiple testing.

Gene names were also searched against the most relevant canine and human literature to look for possible evidences of their functional role in determining key phenotypical traits.

Finally, a subset of hits was selected retaining only the genes identified as outlier by: 1) multiple methods; 2) a single method, but falling in a significantly enriched ontology category; or 3) a single method, but being described in the literature to have a significant role in phenotypic development.

## Results

### Data filtering and marker selection

After removing loci mapping on chromosomes X and Y and following genotyping and quality cleaning steps performed in SVS, both per sample and per locus, we retained the 379 samples that were all successfully genotyped with call rate > 0.99 at 126,848 autosomal SNPs (73%, hereafter referred to as the 126k dataset). These samples included the 12 CWDs and the 12 Carpathian wolves, plus the 12 German Shepherds and the additional 343 dog genotypes from 30 breeds obtained from the LUPA project dataset. A subset of 57,020 SNPs (33%) was retained after LD pruning at threshold r^2^ = 0.2 (the 57k dataset). Finally, a smaller set of 9,063 SNPs (5.2%) was obtained after discarding all sites with any missing data (the 9k dataset).

### Summary statistics

In a pairwise *F*_ST_ matrix of the genetic distances among groups (Additional file 1: Figure S1) computed from the 126k dataset, CWDs were relatively divergent from Carpathian wolves (*F*_ST_ = 0.33) but, as expected, the breed least differentiated from German Shepherds (*F*_ST_ = 0.19).

We found considerable genome-wide variability within groups (Additional file 2: Fig. S2a). Overall, heterozygosity was generally higher in dogs (*H*_o_ = 0.265 ± 0.032) than in wolves (*H*_o_ = 0.231 ± 0.025. However, a direct comparison between wolves and dogs should be treated with caution due to the possible ascertainment bias from the SNP array, mostly designed on dogs, although it is expected to be minimal when considering closely related taxa [30]. CWDs showed heterozygosity levels (*H*_o_ = 0.249) lower than most breeds but, as expected, slightly higher than in German Shepherds (*H*_o_ = 0.234, *p*-values < 0.05 ; *t*-test) and also than Carpathian wolves (*H*_o_ = 0.231, *p*-values < 0.05 ; *t*-test), which showed values coincident with those described in other wolf studies based on SNP chip genotyping (*H*_o_ = 0.210-0.240; [21, 30, 45]).

### Assignment and admixture tests

**Fig. 1:**
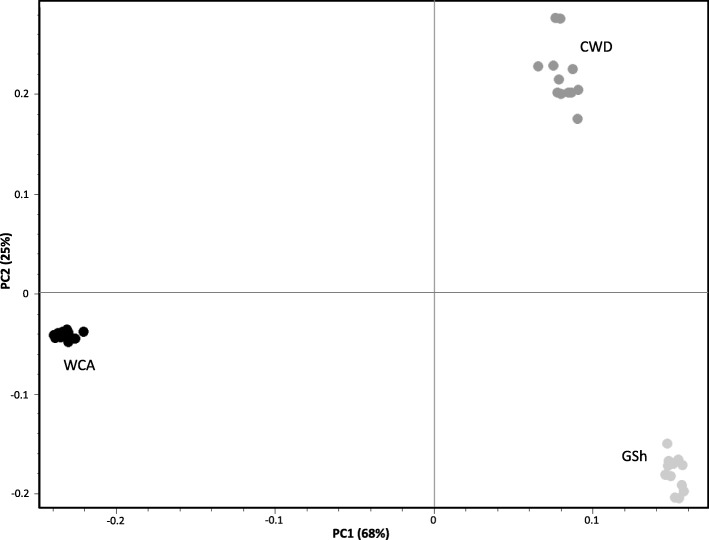
PC1 vs. PC2 results from an exploratory principal component analysis (PCA) computed in SVS on the 126k SNP dataset and including Carpathian wolves (WCA; black dots), German Shepherds (GSh; light grey dots), and Czechoslovakian Wolfdogs (CWD; dark gray dots). The two axes are not to scale, in order to better distinguish individuals along PC2

In an exploratory PCA performed considering CWDs and their parental populations (Fig. 1), the first two axes of the PCA clearly discriminated the three groups, explaining more than the 90% of the whole genetic variability, with Czechoslovakian Wolfdogs plotted along the first axis (which explains 68% of variability) between wolves and dogs, though closer to the latter in accordance to the history of the breed. When we considered the whole 126k dataset (Additional file 3: Figure S3), Czechoslovakian Wolfdogs were located intermediate between German Shepherds and Carpathian wolves along the PC1 axis, which explained more than 30% of the entire genetic variability, and well separated from the other dog breeds overall. Along axis 2, CWDs and German Shepherds clustered close to one another, likely for the higher number of individuals sharing common genetic components compared to those belonging to other breeds, as it occurs when regrouping these same taxa in a neighbor-joining tree [20].

**Fig. 2:**
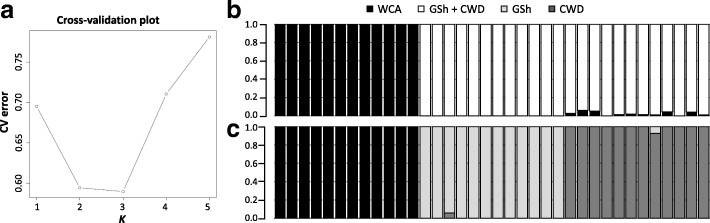
Admixture results obtained running the 57k SNP dataset with with *K* from 1 to 5 and including genotypes from Carpathian wolves (WCA), German Shepherds (GSh) and Czechoslovakian Wolfdogs (CWD). **a** Cross validation plot showing the most likely number of genomic clusters. **b** Admixture results at *K* = 2 show how Carpathian wolves are clearly separated from the two dog groups that cluster together. **c** Admixture results at *K* = 3 show that the three groups are well differentiated from one another

Results from Admixture, run with the 57k dataset and including only CWD, Carpathian wolf and German Shepherd genotypes, showed that the first main decrease in CV error was observed at *K* = 2 (Fig. 2a), when Carpathian wolves (mean estimated membership of population to the assigned cluster *Q*_1_ = 1.00) were clearly separated from the two dog breeds (Fig. 2b), which clustered together (mean *Q*_2_ = 0.987), although several CWDs (*Q*_2_ = 0.975) presented limited but clear traces of wolf components (individual *q*_i_ ranging from 0.940 to 1.00). However, the optimal number of genetic clusters corresponded to *K* = 3 (Fig. 2c), when CWDs (*Q*_3_ = 0.994) were clearly separated from both Carpathian wolves (*Q*_1_ = 1.00) and German Shepherds (*Q*_2_ = 0.995).

**Fig. 3:**
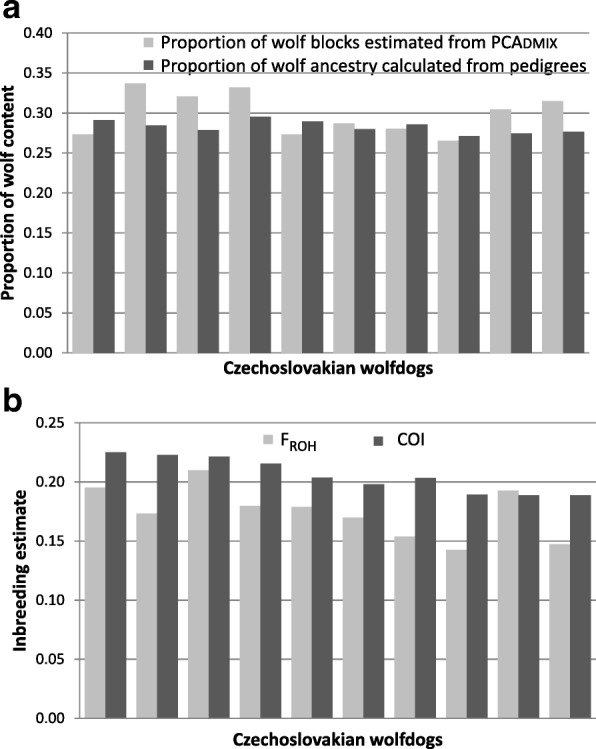
Wolf ancestry proportions and inbreeding rates. **a** Comparison between individual wolf proportions estimated from the analysis of blocks of 10 consecutive, non-overlapping SNPs performed in PCAdmix (in light grey) and individual wolf ancestry rates obtained from pedigrees using BreedMate Pedigree Explorer (in dark grey). **b** Comparison between the individual frequency of ROHs (F_ROH_), calculated in SVS as the proportion of ROHs on the genome length spanned by the analysed SNPs (in light grey), and the individual Wright’s inbreeding coefficient (COI) estimated from the pedigrees with the software U-WGI (in dark grey)

In CWDs, the average genome-wide proportion of blocks assigned by PCAdmix to the reference wolf population was 0.30±0.03, with individual assignment values ranging from 0.27 to 0.34, significantly higher (*p*-values = 1.75 × 10^-10^; *t*-test) than the mean proportion of membership to the wolf cluster (*q*_w_) estimated from Admixture at *K* = 2. Conversely, PCAdmix assignment values were not significantly different (*p*-values = 0.09, *t*-test) from the percentage of wolf ancestry estimated from the pedigrees, whose mean proportion was 0.28±0.01, with individual scores ranging from 0.27 to 0.30 (Fig. 3a).

### Runs of homozygosity, linkage and relatedness

**Fig. 4:**
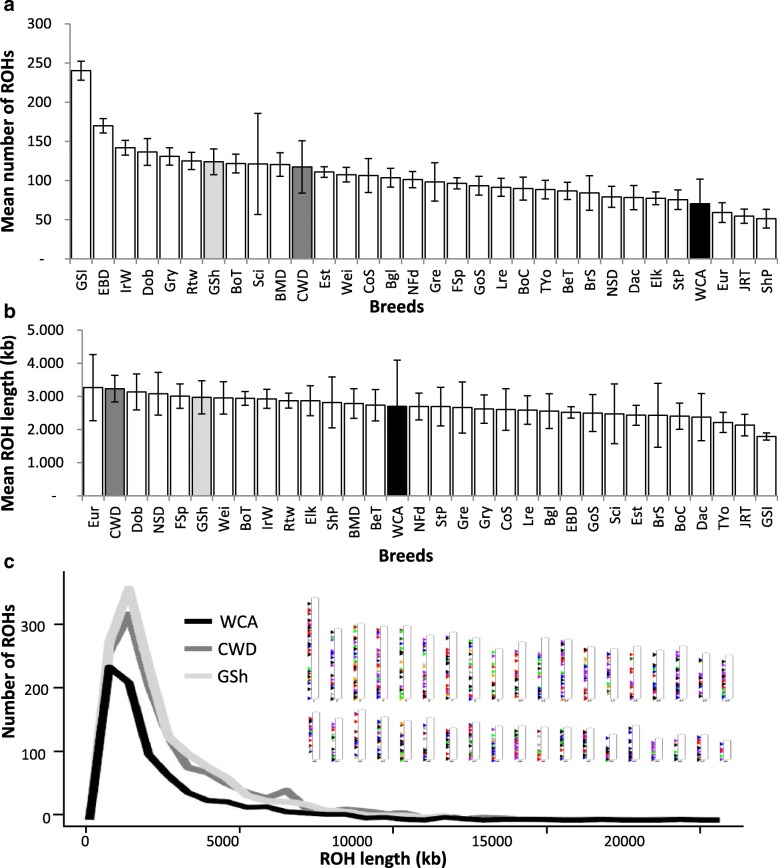
Runs of homozygosity (ROH) analysis. **a** Mean number of ROHs per breed. Czechoslovakian Wolfdogs (CWD) show a mean number of ROHs intermediate between values from parental populations. German Shepherds (GSh) are closer to the breeds with the highest values whereas Carpathian wolves (WCA) to breeds with the lowest values. Bars indicate standard deviations. **b** Mean ROH length (kb) per breed. The mean length of ROHs in Czechoslovakian Wolfdogs (CWD) is wider than parental populations suggesting a high recent inbreeding rate. Bars indicate standard deviations. **c** Distribution of ROH lengths in the three groups. Carpathian wolves (WCA; black line) show most of ROHs of 1000 kb length whereas German Shepherds (GSh; light grey line) and Czechoslovakian Wolfdogs (CWD; dark grey line) exhibit similar patterns, both with most of ROHs around 2000 kb length. However, Czechoslovakian Wolfdogs also show a second peak of ROHs of about 7000 kb length suggesting a stronger inbreeding in more recent generations. Bar plots indicate the 38 Czechoslovakian Wolfdog autosomal chromosomes which show a quite uniformly distributed number of ROHs

Analyzing the whole 126k dataset, CWDs showed a mean number of ROHs (117 ± 33), intermediate between that of German Shepherds (124 ± 16) and that of Carpathian wolves (71 ± 31) (Fig. 4a). As expected according to recent the history of the breed, which allowed a very short time for recombination to break up segments that were identical-by-descent, CWDs showed a mean ROH length (3.234 ± 400 kb) longer than both German Shepherds (2.971 ± 501 kb) and Carpathian wolves (2.699 ± 1.398 kb) (Fig. 4b). This was due to the fact that, although the mode of the ROH length in CWDs and German Shepherds was similar (with most of their ROHs around 2000 kb-long), and much longer than in Carpathian wolves (about 1000 kb), CWDs also showed a second peak of ROHs of 7000 kb length, suggesting that inbreeding events also occurred in the few generations after the breed creation (Fig. 4c).

CWDs showed a mean value of the inbreeding coefficient F_ROH_ (0.17 ± 0.02) similar to German Shepherds (0.16 ± 0.02; *p*-value = 0.10 ; *t*-test) but significantly higher than Carpathian wolves (0.08 ± 0.03; *p*-value < 0.05 ; *t*-test) with individual, F_ROH_ values ranging from 0.14 to 0.21 (Fig. 3b). F_ROH_ was significantly correlated with the inbreeding coefficient estimated from the genotype information *F* (R^2^ > 0.395; *p* < 0.01; Additional file 2: Figure S2b, c) and also with the pairwise coefficient of inbreeding calculated on the basis of pedigree data (COI), that ranged from 0.19 to 0.23 (R^2^ > 0.369; *p* < 0.01; Additional file 4: Figure S4).

**Fig. 5:**
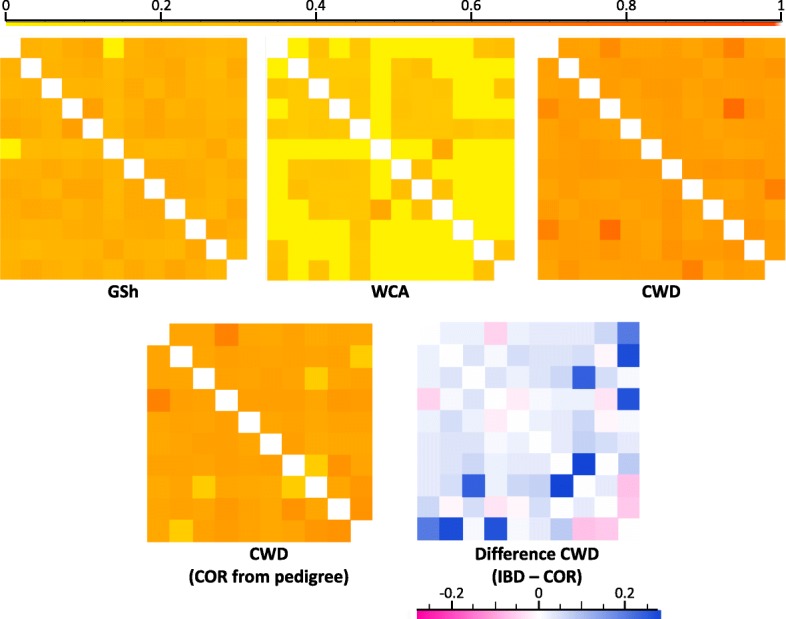
Relatedness analyses. Chromatograms represent pairwise Isolation-by-distance (IBD) scores between Czechoslovakian Wolfdog (CWD), Carpathian wolf (WCA) and German Shepherd (GSh) individuals computed using SVS and CWD coefficient of relatedness (COR) estimated from their pedigrees using the software BreedMate Pedigree Explore. Interestingly, a comparison between the two approaches shows marked differences in some Czechoslovakian Wolfdogs

Looking at identity-by-descent between individuals, the highest mean values of pairwise IBD scores (*p*-values < 0.05 ; *t*-test), as expected according to the low number of founders used in the first steps of the breed creation, were observed in CWDs (0.477 ± 0.049, ranging from 0.426 to 0.738), followed by German Shepherds (0.362 ± 0.054, ranging from 0.000 to 0.451) and then by Carpathian wolves (0.112 ± 0.034, ranging from 0.000 to 0.403). The IBD values found in CWDs were highly concordant (R^2^ = 0.584; *p* < 0.01) with the coefficients of relatedness (COR) estimated from the pedigrees (mean 0.431 ± 0.040, ranging from 0.380 to 0.607), though the pairwise scores between individuals detected from the two approaches in some cases showed marked differences (Fig. 5).

The mean LD in CWDs was intermediate (r^2^ = 0.26) between German Shepherds (r^2^ = 0.30) and Carpathian wolves (r^2^ = 0.13). Similarly the LD decreased to values of r^2^ < 0.10 at a smaller distance in Carpathian wolves (18 kb) than in CWDs (76 kb) and German Shepherds (110 kb; Additional file 5: Figure S5).

### Demographic trends and admixture timing in Czechoslovakian Wolfdogs

**Fig. 6:**
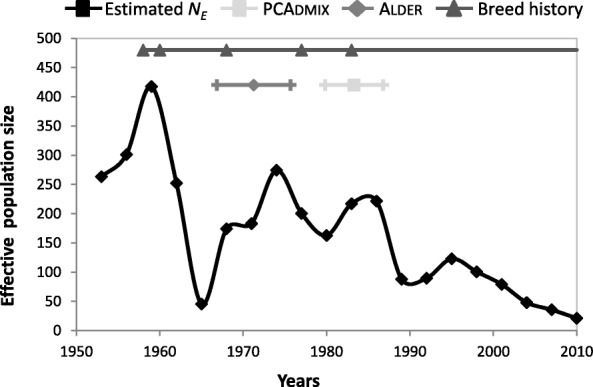
Estimates of demographic trends. The effective population size *N*_*E*_ estimated from LD (squares on black line) shows a decreasing trend over time, though it shows four growth peaks that are concordant with the deliberate crossings with wolves that occurred in the history of the breed (triangles on the dark grey line). The temporal distribution of the admixture events deduced from PCAdmix (squares on light grey horizontal bars) and the time intervals reconstructed by Alder (diamonds on grey horizontal bars) are also described*.* Square, triangle and diamond symbols represent mean values whereas vertical sticks represent confidence intervals

The demographic trajectory estimated from LD well-reflected the history of the breed, which experienced a continuous population decline begun 20 generations ago, thus in the late 1950s’, ranging from a maximum of 418 individuals in 1959 to a minimum of 21 individuals in 2010 (Fig. 6). The only four growth peaks in *N*_*E*_ were observed in periods corresponding to the deliberate crossings with wolves performed for the creation of the breed, plus another moderate one in more recent times not matching any registered crossing.

The software Alder [39] identified significant admixture between the parental populations (*p*-values = 1.0 × 10^-17^) in our CWDs, with successful decay rates (meaning that both the parentals could have been fully sampled; [39]). Hybridization was estimated to have occurred about 12.91 ± 1.47 generations before sampling, which, assuming a wolf generation time of 3 years [36], corresponded to a period ranging from 1967 to 1976, centred around 1971 (Fig. 6).

Results from PCAdmix, used to estimate individual admixture times, showed that the individual number of switches from German Shepherd to Carpathian wolf ancestry blocks ranged from 165 to 367 (mean value 196 ± 55), indicating that the admixture likely occurred from 7.8 to 10.1 generations before individual sampling. Considering the same value of 3 years per generation [36], when converted into years these values indicated that the oldest individual hybridization event likely traced back to 1975, whereas the most recent one traced to 1990, highlighting slightly more recent times than those provided by the software Alder.

### Estimating wolf and dog local genome ancestry, gene search and gene ontology

The analysed CWDs revealed a complex genomic mosaic of wolf and dog ancestry, as reconstructed by PCAdmix (Additional file 6: Figure S6).

From the 10-SNP blocks found to be fixed for wolf or dog haplotypes in all CWDs by PCAdmix, we identified 14 “wolf-like” blocks, including 31 protein-coding genes significantly enriched for metabolic and enzymatic processes and for HP categories related to aortic and renal disorders, and 1784 “dog-like” blocks, including 2238 annotated protein-coding genes, significantly enriched for GO categories mainly related to brain and heart development (Table 1 and Additional file 7: Tables S1a-S1d).

**Table 1:** Subset of wolf-like (a) and dog-like (b) outlier genes detected in Czechoslovakian Wolfdogs analysed in this study which have been previously described in the canid literature

Gene name	Methods	Chr	Start (bp)	End (bp)	Reference	Association
a						
*CRHBP*	BayeScan	3	29,726,312	29,738,001	[60]	Social behavior and maternal aggression
*NPHP4*	PCAdmix, GO	5	59,805,955	59,936,808	[64]	Bone and retinal disorder
*ENO1*	PCAdmix	5	62,301,164	62,312,161	[57]	Related to mRNA transcript variants, genes responsible for bone and cartilage tissues
*ASTN2*	BGC	11	70,248,612	70,977,896	[57]	Related to mRNA transcript variants, genes responsible for bone and cartilage tissues
*PCDH15*	BGC, GO	26	33,962,360	34,571,935	[63]	Vision regulation and hearing abilities
*BMP3*	BGC	32	5,207,833	5,231,966	[58]	Morphological features: paws and bones
b						
*ARID1B*	PCAdmix	1	46,370,636	46,799,104	[68]	Cellular responses, DNA repair
*URI1*	BGC, GO	1	121,528,137	121,612,185	[57]	DNA-binding
*RPE65*	*F*_ST_ SNP	6	76,887,399	76,911,146	[64, 67]	Dog diseases (Leber congenital amaurosis)
*EPAS1*	PCAdmix, GO	10	48,551,410	48,634,643	[77]	Environmental adaptation
*ASCC3*	PCAdmix, GO	12	58,592,025	58,932,720	[68]	Cellular responses, DNA repair
*GRIK2*	PCAdmix, GO	12	59,590,231	59,992,091	[68]	Lipid metabolism
*SMARCD3*	PCAdmix, GO	16	15,279,418	15,289,275	[77]	Muscle cell differentiation, heart morphogenesis
*ZMAT4*	*F*_ST_ SNP, PCAdmix	16	24,561,867	24,889,045	[57]	DNA-binding
*ADAM9*	PCAdmix , GO	16	26,410,907	26,551,122	[64]	Dog diseases (cone-rod dystrophy)
*STRN*	*F*_ST_ SNP	17	29,273,978	29,365,239	[78]	Dog diseases (arrhythmogenic right ventricular cardiomyopathy)
*MGST2*	PCAdmix	19	3,067,163	3,070,563	[68]	Cellular responses, DNA repair
*NOCT*	PCAdmix, GO	19	3,589,720	3,607,191	[65]	Circadian rhythms, body weight and digestion
*SLC7A11*	PCAdmix	19	4,289,915	4,371,635	[68]	Lipid metabolism
*CNTN5*	PCAdmix	21	1,128,048	1,614,989	[20]	Nervous system differentation
*OXT*	PCAdmix	24	18,193,429	18,194,002	[69]	Learning and memory processes
*CBDs*	PCAdmix	24	20,614,030	20,971,219	[68]	Immune system
*DEFB119*	PCAdmix	24	20,905,210	20,918,355	[68]	Immune system
*HM13*	PCAdmix, GO	24	21,026,827	21,067,920	[68]	Cellular responses, DNA repair
*RALY*	ROH	24	23,211,141	23,262,511	[46]	Coat color
*ASIP*	ROH	24	23,354,642	23,393,918	[20, 46]	Coat color, social behavior
*NCOA6*	ROH	24	23,802,887	23,866,792	[68]	Co-activation of several hormone-dependent receptors
*ACSS2*	ROH	24	23,928,670	23,972,633	[68]	Lipid metabolism
*TMEM132D*	*F*_ST_ SNP, PCAdmix	26	2,074,728	2,662,470	[68]	Oligodendrocyte differentiation, metabolism
*CUX2*	PCAdmix, GO	26	8,730,082	8,996,271	[68]	DNA-binding
*SEZ6L*	PCAdmix, GO	26	19,889,395	20,079,319	[70]	Social behavior
*ARVCF*	BGC, PCAdmix, GO	26	29,314,144	29,534,294	[70]	Polydactyly and morphological features
*COMT*	PCAdmix, GO	26	29,360,372	29,366,006	[70]	Social behavior (aggression and attention regulation)
*PCDH15*	BayeScan, PCAdmix, *F*_ST_ SNP, GO	26	33,962,360	34,571,935	[63]	Polydactyly and morphological features, vision and hearing abilities, communication and behavior
*BMPR1B*	PCAdmix, GO	32	17,819,265	17,978,113	[66]	Polydactyly and morphological features
*UNC5C*	*F*_ST_ SNP, PCAdmix, GO	32	17,987,785	18,332,959	[66]	Tumor suppression
*BANK1*	PCAdmix, GO	32	23,281,315	23,603,279	[67]	Regulation processes of calcium ions
*TGIF1*	PCAdmix	32	32,950,116	32,950,934	[66]	Nervous system differentation
*IGF2BP2*	PCAdmix	34	18,368,131	18,522,156	[75, 76]	Lipid metabolism
*MARCH7*	BGC	36	5,499,129	5,531,823	[43]	Cellular responses, DNA repair
*NHEJ1*	PCAdmix, GO	37	25,633,562	25,719,307	[80]	Dog diseases (Collie eye anomaly)
*SLC4A3*	PCAdmix	37	26,136,624	26,149,312	[64, 79]	Dog diseases (progressive retinal atrophy)

When we considered ROHs that were shared by all Czechoslovakian Wolfdogs, we identified a genomic region of about 15 Mb on Chr24 that was always assigned as dog-derived by PCAdmix. This region hosted 29 annotated protein-coding genes, including the coat color regulating genes *ASIP* and *RALY* [20, 46], and genes significantly enriched for a high number of HP categories linked to amino acid metabolism (Table 1 and Additional file 7: Tables S2a-S1b).

Based on the lowest *F*_ST_ between Czechoslovakian Wolfdogs and Carpathian wolves, we identified 15 wolf-like SNPs and one 10-SNP block on chr24 that hosted 1 gene included in significantly enriched GO and HP categories principally related to regulation of catabolic processes, response to external stimulus, locomotory and learning disability (Table 1 and Additional file 7: Tables S3a-S3b; S4a-S4b). When we considered the lowest *F*_ST_ between Czechoslovakian Wolfdogs and German Shepherds, we identified 241 dog-like SNPs and 9 dog-like blocks of 10 consecutive SNPs that included 25 annotated protein-coding genes, significantly enriched for BP category mainly related to palate development and GO categories principally related to regulation of ion transmembrane transport (Table 1 and Additional file 7: Tables S3c-S3d; S4c-S4d).

BGC results detected 78 SNPs with an excess of wolf ancestry (significantly negative values of α) and 62 SNPs with an excess of dog ancestry (significantly positive values of α), with overall higher absolute values in the latter (Additional file 8: Figure S7a). The 50-kb regions surrounding the SNPs with excess of wolf ancestry contained 109 coding genes enriched for HP categories mainly related to cerebral atrophy (Table 1 and Additional file 7: Tables S5a-S5b). Conversely, regions surrounding the SNPs with excess of dog ancestry contained 79 protein-coding genes that were mostly enriched for a GO biological process related to granulocyte regulation, and HP categories linked to earlobe morphology and skeletal, aortic or parathyroid disorders (Table 1 and Additional file 7: Tables S5c-S1d).

Finally, comparing CWDs with German Shepherds, BayeScan identified 29 outlier SNPs with positive α values (suggestive of diversifying selection) hosted in regions including 29 protein-coding genes, significantly enriched for GO categories mainly linked to biological processes such as maternal aggressive behavior and corticotropin secretion, and HP categories principally related to abnormal proportions of face and hands (Table 1 and Additional file 7: Tables S6a-S6b). When we compared CWDs to Carpathian wolves, BayeScan identified 7 outlier SNPs with positive α values that were hosted in regions including 7 annotated protein-coding genes, significantly enriched for GO categories mostly linked to tRNA regulation (Table 1 and Additional file 7: Tables S6c-S6d).

## Discussion

The fast-growing number of registered Czechoslovakian Wolfdogs worldwide demonstrates the elevated economical value of this breed and the need of a deeper comprehension of the genetic bases of its morphological and behavioural traits, as well as of the causative mutations of some common diseases. In this study we provide the most complete genomic description of the breed to date by genotyping 12 individuals at 170k SNPs and comparing their genome-wide diversity to samples as representative as possible of their parental populations (Carpathian wolves and German Shepherds) and to genomic profiles from 30 other common breeds publicly available from the LUPA project [17, 20].

From a preliminary genomic screening, based on pairwise *F*_ST_ values, multivariate and assignment procedures, CWDs appeared highly differentiated from all the other analysed breeds and were also well-distinguished from both parental populations. In particular, despite our limited sampling, the Bayesian clustering analysis performed in Admixture revealed the presence of three optimal clusters clearly separating CWDs from both parental populations, consistent with previous findings based on a few autosomal microsatellites [3, 5–7].

Compared with the LD-based approach of Admixture (*K* = 2), the PCA-based admixture deconvolution approach implemented in PCAdmix [27], which reflects the ancestry proportions of an individual better than Admixture [28], identified larger wolf components (> 25%) in the genome of the analysed CWDs. These proportions compared well with the pedigree-based estimates, confirming that such a haplotype block-based approach is an appropriate and reliable tool to assess real admixture proportions from genomic data [28].

Our results on the observed genome–wide heterozygosity levels in CWDs were consistent with other studies, based on different types and number of markers [5, 7, 20]. In particular, values of autosomal heterozygosity in our small sample of CWDs were slightly higher than those observed in the parental populations, consistent with the recent admixture occurred in the creation of the breed [3, 5, 6] that is still visible in the large genomic regions hosting both dog and wolf haplotype blocks, thus representing islands of high heterozygosity, even after *c.* 30 generations since the breed foundation and *c.* 11 generations since the last official outcrossing, contrasting the expected decay in heterozygosity due to inbreeding.

On the contrary, the lower heterozygosity observed in Carpathian wolves, which was expected to be higher than in dogs for genomic sequences [47], should be treated with caution, since it could be partially attributable to a possible ascertainment bias linked to the original SNP chip design, mostly based on dog variation [30, 48], although such event is unlikely for closely related taxa diverging less than one million years [30]. However, our estimates of observed heterozygosity in Carpathian wolves well compare with those from other Central-Eastern European wolf populations reported in previous studies using the same SNP chip approaches [21; 30; 45] and certainly did not affect the ability of our methods to discriminate between wolf-like and dog-like haplotype blocks in CWDs.

The analysis of ROHs allowed us to better reconstruct the breed history and clarify its dynamics. Czechoslovakian Wolfdogs showed a higher number of long ROHs (> 5 Mb) than the progenitors, reflecting the recent inbreeding events [49–51] that occurred during and after the origin of the breed. Moreover, coherently with the low number of founders utilized in the breed creation, CWDs showed inbreeding coefficient values (F_ROH_) higher than both parental populations [3], and also higher values of relatedness between individuals, on average.

Though a direct comparison between genomic data and pedigree information should be treated with caution given the different methodologies these two types of computations rely on [33], estimates of inbreeding levels calculated from the frequency of homozygosity regions (F_ROH_) were comparable with those calculated from the coefficient of inbreeding (COI) derived from the available pedigree data. Such a concordance confirms the reliability of several proxies in identifying inbreeding, which is crucial for breeders since matings among closely related individuals can affect their offspring fitness due to the increased probability of deleterious alleles being expressed in their phenotypes. Conversely, in several cases the coefficient of relatedness (COR) between individuals estimated from the pedigrees underestimated the IBD (identity-by-descent) scores determined from genetic profiles. Such discrepancies could be due to the higher ability of genome-wide methods to identify random segregation effects compared to pedigree-based methods [33], or to the uncertainties of pedigree records, in which breeders might deliberately not report some crossings between related individuals, since the possible negative effects on health could reduce the marketability of dogs [51], even if this latter possibility appears very unlikely given the strict breeding control operated by the military during the early years of breed establishment.

Therefore, genomic reconstructions represent a useful tool to implement carefully planned mating strategies among breeders in order to predict and contrast possible deleterious effects such as lethal genetic disorders, reduction of fertility, and lower adaptive potential [52, 53]. For these reasons, genomic pairwise IBD values and ROH-based metrics could provide breeders with additional information that could be evaluated for the selection of lineages to reduce the levels of inbreeding per generation, taking into account not only the blood lines but also the stochastic effects of recombination [31, 33].

Our genome-wide characterization allowed us to verify the timing of the admixture in the cohort of the analysed CWDs, which compared well with the key steps of the breed selection, namely the repeated insertion of wolf alleles that officially continued until 1983. When applying Alder, hybridization was estimated to have occurred from 1967 to 1976, roughly corresponding to the midpoint of the known crossing events, whereas PCAdmix better identified the most recent ones. These findings show that genomic-based dating methods can be effective and complementary in tracing recent hybridization events both in hybrid breeds such as CWD and in wild-living populations [16].

The *N*_*E*_ trends estimated from the LD patterns showed that, despite the growing number of registered individuals, *N*_*E*_ overall declined from the breed origin to the present. This decreasing trend is likely due to the progressive artificial selection and to the so-called “popular sire effect”, namely the overrepresentation of the genetic contribution of popular dogs (e.g. small number of winner individuals at dog shows) in subsequent generations of the breed [54]. Conversely, *N*_*E*_ fluctuations, with four main peaks around years 1959, 1968, 1974 and 1986, are consistent with the official wolf *x* dog registered crossings (1960, 1968, 1974 and 1983). However, we unexpectedly detected an additional slight increase in *N*_*E*_ around 1995, which could be due to the genetic contribution from a distinct lineage [54] of CWDs (e.g. from the Slovakian to the Czech lineage), or might be the signal of an undeclared wolf contribution that occurred after the official breed recognition. Should this second hypothesis be confirmed, it would value genomic investigations also as a tool to identify illegal crossings of wild species protected under the CITES Convention with commercialised domestic breeds [55, 56]. Nonetheless, this overall, fast decline in *N*_*E*_ did not erode all the additional variation provided by the wolf founders, since the heterozygosity levels appear to be still currently slightly higher in the analysed CWDs than in German Shepherds.

Looking at the genomic landscape of Czechoslovakian Wolfdogs, PCAdmix results showed a variegated chromosomal ancestry mosaic, ranging from fully dog-derived to mostly wolf-like regions. A gene search based on ancestry-outlier regions obtained from multiple methods, which was possible thanks to the availability of the well-annotated dog reference genome, allowed us to identify more than 300 genes with an excess of wolf ancestry and more than 2000 genes with an excess of dog ancestry in Czechoslovakian Wolfdogs compared to random expectations.

The key wolf-like genes we identified were mainly related to body size and shape traits, which could explain the overall morphological similarity of CWDs with wolves. In particular, we detected two wolf-excess genes, *ASTN2* and *ENO1*, which were described in the human genome to be adjacent to loci putatively responsible for bone and cartilage tissue production and that were earlier found to be under selection in European wolves [57]. Another 9 wolf-like genes were related to key morphological features, such as prominent occiput (*ITCH*) and prominent nasal bridge (*CLIP1*, *WDPCP*), narrow face (*AP4M1*, *CLIP1*), short ears (*CAMTA1*), narrow and small mouth (*KCNAB2*, *CAMTA*, *AP4M1*, *CLIP1*), pointed chin (*CLIP1*, *AP4M1*), strong facial musculature (*CLIP1*, *AP4M1*, *HNRNPA2B1*), robust paws and bones (*AGGF1*, *BMP3*; [58]), all typical of the breed. However, other wolf-excess genes were described to be associated with communication and behaviour. In particular, *CRHBP*, coding for Corticotropin Releasing Hormone Binding Protein, is a gene expressed during pregnancy [59], involved in the anomalous maternal aggressive behavior against puppies observed both in mice and in Australian Working Kelpie female dogs [60]. Such peculiar behaviour is well known also in Czechoslovakian Wolfdogs, where mothers killing their offspring shortly after parturition have often been observed (A. Camatta, personal communication). *PCDH15* has been identified as a candidate gene related to echolocation in mammals [61, 62] and has been described to be under selection in different ecological contexts in wolves [63]. Similarly, other wolf-excess genes were related to cardiac (*KCNAB2*, *WDPCP*), pancreatic (*PLCG2*), bone and retinal (*NPHP4*) disorders that have been widely described in a number of dog breeds [64], but not yet in wolves, and that could provide a higher resistance of CWDs to such disorders compared to German Shepherds.

Conversely, a number of behavioral traits desired by the breeders could be hosted in a large set of dog-like genes, often involved in brain development, which has been demonstrated to be a pivotal target of domestication [65]. In particular, two genes were related to neural differentiation and formation of the nervous system (*TGIF1* and *CNTN5*; [20, 66]) and the gene *TMEM132D* was involved in oligodendrocyte differentiation that was previously identified in dog and wolf selection scans [57, 67, 68]. Similarly, we identified a number of dog-like genes playing important roles in learning and memory processes, such as *OXT*, which can affect canine cognition, tolerance, adaptation and maternal behaviour [69], in vision and hearing abilities, such as *PCDH15* [63], and in the regulation of circadian rhythms, body weight and digestion, such as *NOCT* [65, 68], which could be crucial in adapting the physiological activity of CWDs to that of their human owners.

Interestingly, we also detected genes described to be correlated with sociability: *COMT*, a gene involved in dopamine catalysis and in regulating aggressive behaviors and attention in many breeds, and *SEZ6L*, both mapping on chr26 and described as significantly associated with the time dogs spend in close proximity of humans [70], reinforcing the hypothesis that the transformation of negative defensive reactions toward humans into positive responses could have been a primary step in early dog domestication [70] and that deliberate artificial selection on tameness may been have further reinforced[65]. Direct or indirect artificial selection for tameness or sociability played a key role on the evolution of a number of other domesticated and wild taxa: the possibility of a strong artificial selection on tameness was demonstrated also in the rat (*Rattus norvegicus*; [71]) and in the red junglefowl (*Gallus gallus*; [72]), showing that a number of other traits were influenced by their sole selection on tameness, as already revealed in the keystone study by Balyaev and colleagues on silver foxes [11], leading to the concept of General Domestication Syndrome to indicate a set of phenotypic traits common to a number of domesticated species [73]. However, the long-lasting presence of human-dominated landscapes can indirectly affect the genetic bases of tameness also in wild-living populations, such as the Apennine brown bear (*Ursus arctos marsicanus*), which shows reduced aggressive behaviours compared to other populations reflected in a unique genomic signature [74].

Another set of dog-excess genes were involved in the regulation of calcium ions (*BANK1*), in the co-activation of several hormone-dependent receptors (*NCOA6*) and in DNA-binding (*CUX2*, *URI1*, *ZMAT4*), which were also identified to be under selection in previous canid studies [57, 67, 68].

Additionally we identified other dog-like genes known to be involved in immune functions, such as those coding for the immunity-related beta-defensins (*CBDs* and *DEFB119*) and those responsible for cellular responses and DNA repair (*ARID1B*, *ASCC3*, *HM13*, *MGST2*, *MARCH7*), and tumor suppression (*UNC5C*), that were identified to be hosted in key-differentiating regions for dog domestication [68]. We detected another four dog-excess genes, *IGF2BP2* [75, 76] and *SLC7A11*, *ACSS2*, *GRIK2* [68], which were related to lipid metabolism and to the synthesis of energy that could indicate the importance of dietary modifications during the domestication process, especially during the phase of breed creation [68].

We also found two genes (*ASIP* and *RALY*) involved in regulating coat coloration by the synthesis of the yellow pigment known as pheomelanin, that could confer the typical color to the breed [20, 46]. Interestingly, recent evidences demonstrated that variations in *ASIP*, found to be under selection also in ancient Asian dog breeds [77], can influence social behavior too, most likely through its antagonistic effects on melanocortin receptors or α-melanocortin stimulating hormone [68, 77], confirming that morphological and behavioral characteristics in canids can be strongly linked [11, 14].

However, we identified also a series of dog-excess genes previously described in the literature to be linked to a number of common dog disorders such as arrhythmogenic right ventricular cardiomyopathy (*STRN*; [78]), progressive retinal atrophy (*SLC4A3*; [64, 79]), Collie eye anomaly (*NHEJ1*; [80]), cone-rod dystrophy (*ADAM9*; [64]), and canine Leber congenital amaurosis, previously known as congenital stationary night blindness (*RPE65*; [64]), that are typical of the parental population German Shepherd and that could be retained during the strong artificial selection that occurred during the CWD creation.

## Conclusions

Our study provides the first genome-wide characterization of the Czechoslovakian Wolfdog, highlighting how the breed, despite the declared low number of founders, currently shows relatively high levels of heterozygosis thanks to its hybrid ancestry. Our genome-wide approach confirmed to be a valid method in reconstructing the breed history and dating its dynamics, to assess the actual wolf ancestry proportions of single individuals, as well as their relatedness. Therefore it could provide a valid instrument also for forensic applications in order to unmask possible trades of individuals sold as purebreds but that originated from illegal crossings with wild wolves, which would be difficult to identify through multivariate and Bayesian assignment procedures based on a limited number of loci or on their morphology alone. Moreover, our gene search approach, made possible by the availability of a well-annotated reference genome, allowed us to identify a first set of genes whose expression and interaction would likely determine the typical wolf-like appearance of the breed. Interestingly, most of the genes associated with brain functions, behaviour, metabolism and disorders we detected are clearly dog-derived, as expected in a breed that, despite its recent hybrid origins, mostly shows typical dog-like phenotypes. The best example is represented by the *COMT* gene, which has been described as the candidate gene for sociability in dogs [70] and only its dog alleles have been retained in the gene pool of the analysed CWDs.

However, finding the causal mutations for single traits needs further research, in particular for polygenic traits [81]. Future genotyping of a larger number of individuals with certified pedigrees from different lineages sampled worldwide will contribute to a deeper comprehension of many genetic, morphological, and behavioural characteristics of this breed. The optimization of a small and rapid marker panel, for example of 96 SNPs, including also mutations for common diseases or particular behaviours, could help to monitor the health of all the commercialized captive-born individuals and to allow their genomic identification, contrasting unreported crossings and illegal trading of wild wolves.

## Additional files

Additional file 1: **Figure S1.** *F*_ST_ heat plot matrix of the genetic distances among groups computed from the 126k dataset in SVS. The most distant breed to Carpathian wolves (WCA) is the English Bulldog (EBD) while the closest one is the ancient breed Shar-Pei (ShP). As expected the least differentiated breed from the Czechoslovakian Wolfdog (CWD) is the German Shepherd (GSh). (PDF 200 kb)

Additional file 2: **Figure S2.** Genetic variability indexes computed in SVS using the 126k SNP dataset. **a** Mean values of observed heterozygosity (*H*_o_) within groups. Czechoslovakian Wolfdogs (in dark gray) show higher levels of heterozygosity than parental populations (Carpathian wolves in black and German Shepherds in light grey), as expected from the recent crossings that originated the breed, but lower than most breeds. Bars indicate standard deviations. **b** Plots of the mean inbreeding coefficient *F* per breed. Czechoslovakian Wolfdogs show a mean *F* value intermediate among the other breeds but lower than both parental populations. **c:** from left to right: individual *F* values for Carpathian wolf (black histograms), German Shepherd (light grey histograms) and Czechoslovakian Wolfdog (dark gray histograms) groups. Bars indicate standard deviations. (PDF 94 kb)

Additional file 3: **Figure S3.** PC1 vs. PC2 results from an exploratory principal component analysis (PCA) computed in SVS on the 126k SNP dataset and including dogs from 30 pure breeds (extrapolated from the available LUPA project dataset; top side of the graph, in grey inside the circle), Carpathian wolves (WCA; black dots to the left), German Shepherds (GSh; light grey dots in the bottom), and Czechoslovakian Wolfdogs (CWD; dark gray dots in the bottom). The two axes are not to scale, in order to better distinguish individuals along PC2. (PDF 202 kb)

Additional file 4: **Figure S4.** Comparison between the individual frequency of ROHs (F_ROH_), calculated in SVS as the proportion of ROHs on the genome length spanned by the analysed SNPs (on the horizontal axis), and the individual Wright’s inbreeding coefficient (COI), estimated from the pedigrees with the software U-WGI (on the vertical axis). The two inbreeding indexes are significantly (p < 0.01) correlated. (PDF 71 kb)

Additional file 5: **Figure S5.** Linkage disequilibrium (LD) decay plot. The vertical axis indicates the mean Estimated R-squared (r^2^), and the horizontal axis indicates the distance in kb at which LD decays. (PDF 220 kb)

Additional file 6: **Figure S6.** Graphical representation, for each chromosome of each analysed Czechoslovakian Wolfdog, of the ancestry components identified by PCAdmix based on the analysis of 10-SNP haplotype blocks. Each horizontal bar represents the two homologous chromosomes of an individual showing in black the genomic regions assigned as wolf-like and in light grey those assigned as dog-like. (PDF 10810 kb)

Additional file 7: **Table S1a.** Wolf-excess genes surrounding outlier wolf-like SNPs (OWS) from PCAdmix. **Table S1b.** Enrichment in gene ontology categories (EGO) of wolf-excess genes surrounding the OWS from PCAdmix. **Table S1c**. Dog-excess genes surrounding outlier dog-like SNPs (ODS) from PCAdmix. **Table S1d.** EGO of dog-excess genes surrounding ODS from PCAdmix. **Table S2a.** Dog-excess genes included in genomic regions within ROHs. **Table S2b.** EGO of the dog-excess genes included in genomic regions within ROHs. **Table S3a.** Wolf-excess genes surrounding OWS from the lowest *F*_ST_ between CWDs and Carpathian wolves. **Table S3b.** EGO of wolf-excess genes surrounding OWS from the lowest *F*_ST_ between CWDs and Carpathian wolves. **Table S3c.** Dog-excess genes surrounding ODS from the lowest *F*_ST_ between CWDs and German Shepherds. **Table S3d.** EGO of dog-excess genes surrounding ODS from the lowest *F*_ST_ between CWDs and German Shepherds. **Table S4a.** Wolf-excess genes surrounding the outlier wolf-like 10-SNP blocks identified from the lowest *F*_ST_ between CWDs and Carpathian wolves. **Table S4b.** EGO of the wolf-excess genes surrounding the outlier wolf-like 10-SNP blocks identified from the lowest *F*_ST_ between CWDs and Carpathian wolves. **Table S4c.** Dog-excess genes surrounding outlier dog-like 10-SNP blocks from the lowest *F*_ST_ between CWDs and German Shepherds. **Table S4d.** EGO of dog-excess genes surrounding outlier dog-like 10-SNP blocks from the lowest *F*_ST_ between CWDs and German Shepherds. **Table S5a.** Wolf-excess genes surrounding OWS from BGC *alpha* parameter. **Table S5b.** EGO of wolf-excess genes surrounding OWS from BGC *alpha* parameter. **Table S5c.** Dog-excess genes surrounding ODS from BGC *alpha* parameter. **Table S5d.** EGO of dog-excess genes surrounding ODS from BGC *alpha* parameter. **Table S6a.** Wolf-excess surrounding OWS from BayeScan. **Table S6b.** EGO of wolf-excess genes surrounding OWS from BayeScan. **Table S6c.** Dog-excess genes surrounding ODS from BayeScan. **Table S6d.** EGO of dog-excess genes surrounding ODS from BayeScan. (XLSX 781 kb)

Additional file 8: **Figure S7a.** BGC *alpha* parameter outlier SNPs. Values lower than 0 indicate excess of wolf alleles, values higher than 0 indicate excess of dog alleles. BGC significant outliers are indicated by blue crosses (top or bottom 1% of the empirical distribution of values) and by red dots (95% credibility intervals of 10,000 iterations not including 0). **Figure S7b.** BayeScan outlier SNPs detected comparing differences in allele frequency between Czechoslovakian Wolfdogs and German Shepherds (right) and between Czechoslovakian Wolfdogs and Carpathian wolves (left). The vertical axis indicates mean *F*_ST_ values between populations, and the horizontal axis indicates the logarithm of posterior odds (log(PO)). The vertical line indicates the log(PO) value corresponding to the false discovery rate threshold of 0.05. Loci on the right of this line are putatively under selection. (PDF 312 kb)

## Abbreviations

BeT: Belgian Tervuren
Bgl: Beagle
BMD: Bernese Mountain Dog
BoC: Border Collie
BoT: Border Terrier
BrS: Brittany Spaniel
CITES: Convention on Internationala Trade in Endangered Species
CoS: Cocker Spaniel
CWD: Czechoslovakian Wolfdog
Dac: Dachshund
Dob: Doberman Pinscher
EBD: English Bulldog
Elk: Elkhound
ESt: English Setter
Eur: Eurasian Dog
FSp: Finnish Spitz
GoS: Gordon Setter
GRe: Greyhound
Gry: Golden Retriever
GSh: German Shepherd
GSl: Greenland Sledge Dog
IrW: Irish Wolfhound
JRT: Jack Russell Terrier
LRe: Labrador Retriever
NFd: Newfoundland
NSD: Nova Scotia Duck Tolling Retriever
Rtw: Rottweiler
Sci: Schipperke
ShP: Shar-Pei
StP: Standard Poodle
TYo: Yorkshire Terrier
WCA: Carpathian Wolves
Wei: Weimaraner

## References

[1] Larson G, Piperno DR, Allaby RG, Purugganan MD, Andersson L, Arroyo-Kalin M (2014). Current perspectives and the future of domestication studies. Proc Natl Acad Sci.

[2] Zeder MA (2015). Core questions in domestication research. Proc. Natl. Acad. Sci..

[3] Smetanová M, Černá Bolfíková B, Randi E, Caniglia R, Fabbri E, Galaverni M (2015). From Wolves to Dogs, and Back: Genetic Composition of the Czechoslovakian Wolfdog. PLoS One..

[4] Allendorf FW, F LR, Paul S, K WJ (2001). The problems with hybrids : setting conservation guidelines. Trends Ecol Evol.

[5] Leroy G, Verrier E, Meriaux JC, Rognon X (2009). Genetic diversity of dog breeds: Between-breed diversity, breed assignation and conservation approaches. Anim. Genet..

[6] Randi E, Hulva P, Fabbri E, Galaverni M, Galov A, Kusak J (2014). Multilocus detection of wolf x dog hybridization in Italy, and guidelines for marker selection. PLoS One..

[7] Bigi D, Marelli SP, Randi E, Polli M (2015). Genetic characterization of four native Italian shepherd dog breeds and analysis of their relationship to cosmopolitan dog breeds using microsatellite markers. Animal..

[8] Voorbij AMWY, Leegwater PA, Kooistra HS (2014). Pituitary Dwarfism in Saarloos and Czechoslovakian Wolfdogs is Associated with a Mutation in LHX3. J. Vet. Intern. Med..

[9] Awano T, Johnson GS, Wade CM, Katz ML, Johnson GC, Taylor JF (2009). Genome-wide association analysis reveals a SOD1 mutation in canine degenerative myelopathy that resembles amyotrophic lateral sclerosis. Proc. Natl. Acad. Sci. U. S. A..

[10] Parker HG, Shearin AL, Ostrander EA (2010). Man’s best friend becomes biology’s best in show: genome analyses in the domestic dog. Annu. Rev. Genet..

[11] Trut L, Oskina I, Kharlamova A (2009). Animal evolution during domestication: the domesticated fox as a model. BioEssays..

[12] Kukekova AV, Temnykh SV, Johnson JL, Trut LN, Acland GM, V TS (2012). Genetics of behavior in the silver fox. Mamm. Genome..

[13] Gogoleva SS, I a V, Volodina EV, Kharlamova AV, Trut LN (2011). Explosive vocal activity for attracting human attention is related to domestication in silver fox. Behav. Processes.

[14] Stone HR, McGreevy PD, Starling MJ, Forkman B (2016). Associations between domestic-dog morphology and behaviour scores in the dog mentality assessment. PLoS One.

[15] Hindrikson M, Remm J, Pilot M, Godinho R, Stronen AV, Baltrūnaité L (2017). Wolf population genetics in Europe: a systematic review, meta-analysis and suggestions for conservation and management. Biol Rev.

[16] Galaverni M, Caniglia R, Pagani L, Fabbri E, Boattini A, Randi E (2017). Disentangling Timing of Admixture, Patterns of Introgression, and Phenotypic Indicators in a Hybridizing Wolf Population. Mol Biol Evol.

[17] Lequarré AS, Andersson L, André C, Fredholm M, Hitte C, Leeb T (2011). LUPA: A European initiative taking advantage of the canine genome architecture for unravelling complex disorders in both human and dogs. Vet. J.

[18] Rothschild MF, Plastow GS (2014). Applications of genomics to improve livestock in the developing world. Livest. Sci.

[19] Varshney RK, Terauchi R, McCouch SR (2014). Harvesting the promising fruits of genomics: applying genome sequencing technologies to crop breeding. Dangl JL. PLoS Biol.

[20] Vaysse A, Ratnakumar A, Derrien T, Axelsson E, Rosengren Pielberg G, Sigurdsson S (2011). Identification of genomic regions associated with phenotypic variation between dog breeds using selection mapping. Akey JM. PLoS Genet.

[21] Stronen AV, Jędrzejewska B, Pertoldi C, Demontis D, Randi E, Niedziałkowska M, et al. North-South differentiation and a region of high diversity in European wolves (*Canis lupus*). PLoS One. 2013;8:e76454. 10.1371/journal.pone.0076454 PMC3795770 24146871

[22] Purcell S, Neale B, Todd-Brown K, Thomas L, Ferreira M a R, Bender D (2007). PLINK: a tool set for whole-genome association and population-based linkage analyses. Am. J. Hum. Genet..

[23] Weir BS, Cockerham CC. Estimating F-statistics for the analysis of population structure. Evolution. 1984;38:1358–70. 10.1111/j.1558-5646.1984.tb05657.x 28563791

[24] Novembre J, Stephens M (2008). Interpreting principal component analyses of spatial population genetic variation. Nat. Genet..

[25] Price AL, Patterson NJ, Plenge RM, Weinblatt ME, Shadick NA, Reich D (2006). Principal components analysis corrects for stratification in genome-wide association studies. Nat. Genet..

[26] Alexander DH, Novembre J, Lange K (2009). Fast model-based estimation of ancestry in unrelated individuals. Genome Res..

[27] Brisbin A, Bryc K, Byrnes J, Zakharia F, Omberg L, Degenhardt J (2012). PCAdmix: Principal Components-Based Assignment of Ancestry along Each Chromosome in Individuals with Admixed Ancestry from Two or More Populations. Hum. Biol..

[28] Falush D, Dorp L van, Lawson D. A tutorial on how (not) to over-interpret STRUCTURE/ADMIXTURE bar plots. BioRxiv. 2016;66431. 10.1101/066431. 10.1038/s41467-018-05257-7 PMC6092366 30108219

[29] Boyko AR, Quignon P, Li L, Schoenebeck JJ, Degenhardt JD, Lohmueller KE (2010). A simple genetic architecture underlies morphological variation in dogs. Hoekstra HE. PLoS Biol..

[30] VonHoldt BM, Pollinger JP, Earl DA, Knowles JC, Boyko AR, Parker H (2011). A genome-wide perspective on the evolutionary history of enigmatic wolf-like canids. Genome Res.

[31] McQuillan R, Leutenegger AL, Abdel-Rahman R, Franklin CS, Pericic M, Barac-Lauc L (2008). Runs of Homozygosity in European Populations. Am. J. Hum. Genet..

[32] Curik I, Ferenčaković M, Sölkner J (2014). Inbreeding and runs of homozygosity: a possible solution to an old problem. Livest. Sci..

[33] Kardos M, Luikart G, Allendorf FW (2015). Measuring individual inbreeding in the age of genomics: Marker-based measures are better than pedigrees. Heredity.

[34] Tenesa A, Navarro P, Hayes BJ, Duffy DL, Clarke GM, Goddard ME (2007). Recent human effective population size estimated from linkage disequilibrium. Genome Res..

[35] Hayes BJ, Visscher PM, Mcpartlan HC, Goddard ME. Novel Multilocus measure of linkage disequilibrium to estimate past effective population size. Genome Res 2003;13(4):635–43. 10.1101/gr.387103 PMC430161 12654718

[36] Skoglund P, Gotherstrom A, Jakobsson M, Götherström A, Jakobsson M (2011). Estimation of population divergence times from non-overlapping genomic sequences: examples from dogs and wolves. Mol. Biol. Evol..

[37] Delaneau O, Marchini J, Zagury J-F (2012). A linear complexity phasing method for thousands of genomes. Nat. Methods.

[38] Muñoz-Fuentes V, Marcet-Ortega M, Alkorta-Aranburu G, Linde Forsberg C, Morrell JM, Manzano-Piedras E (2015). Strong Artificial Selection in Domestic Mammals Did Not Result in an Increased Recombination Rate. Mol. Biol. Evol..

[39] Loh PR, Lipson M, Patterson N, Moorjani P, Pickrell JK, Reich D (2013). Inferring admixture histories of human populations using linkage disequilibrium. Genetics..

[40] Johnson NA, Coram MA, Shriver MD, Romieu I, Barsh GS, London SJ (2011). Ancestral Components of Admixed Genomes in a Mexican Cohort. PLoS Genet..

[41] Gompert Z, Buerkle CA (2012). bgc : Software for Bayesian estimation of genomic clines. Mol. Ecol. Resour..

[42] Foll M, Gaggiotti O (2008). A genome-scan method to identify selected loci appropriate for both dominant and codominant markers: a bayesian perspective. Genetics.

[43] Pilot M, Malewski T, Moura AE, Grzybowski T, Oleński K, Kamiński S, et al. Diversifying selection between pure-breed and free-breeding dogs inferred from genome-wide SNP analysis. G3. 2016;6:2285–98. 10.1534/g3.116.029678 PMC4978884 27233669

[44] Reimand J, Arak T, Adler P, Kolberg L, Reisberg S, Peterson H (2016). g:Profiler-a web server for functional interpretation of gene lists (2016 update). Nucleic Acids Res..

[45] Pilot M, Greco C, vonHoldt BM, Randi E, Jędrzejewski W, Sidorovich VE, Konopiński MK, Ostrander EA, Wayne RK (2018). Widespread, long-term admixture between grey wolves and domestic dogs across Eurasia and its implications for the conservation status of hybrids. Evol. Appl..

[46] Dreger DL, Parker HG, Ostrander EA, Schmutz SM (2013). Identification of a mutation that is associated with the saddle tan and black-and-tan phenotypes in Basset Hounds and Pembroke Welsh Corgis. J. Hered..

[47] Wang G-D, Zhai W, Yang H-C, Wang L, Zhong L, Liu Y-H (2016). Out of southern East Asia: the natural history of domestic dogs across the world. Cell Res..

[48] Gopalakrishnan S, Samaniego Castruita JA, Sinding M-HS, Kuderna LFK, Räikkönen J, Petersen B (2017). The wolf reference genome sequence (*Canis lupus lupus*) and its implications for *Canis* spp. population genomics. BMC Genomics.

[49] Ferenčaković M, Hamzić E, Gredler B, Solberg TR, Klemetsdal G, Curik I (2013). Estimates of autozygosity derived from runs of homozygosity: Empirical evidence from selected cattle populations. J. Anim. Breed. Genet..

[50] Kim ES, Sonstegard TS, Van Tassell CP, Wiggans G, Rothschild MF (2015). The relationship between runs of homozygosity and inbreeding in Jersey cattle under selection. PLoS One.

[51] Iacolina L, Stronen AV, Pertoldi C, Tokarska M, Nørgaard LS, Muñoz J (2016). Novel graphical analyses of runs of homozygosity among species and livestock breeds. Int. J. Genomics.

[52] Bjelland DW, Weigel KA, Vukasinovic N, Nkrumah JD (2013). Evaluation of inbreeding depression in Holstein cattle using whole-genome SNP markers and alternative measures of genomic inbreeding. J. Dairy Sci..

[53] Stronen AV, Salmela E, Baldursdó Ttir BK, Berg P, Espelien IS, Jä Rvi K (2017). Genetic rescue of an endangered domestic animal through outcrossing with closely related breeds: A case study of the Norwegian Lundehund. PLoS One..

[54] Dreger DL, Rimbault M, Davis BW, Bhatnagar A, Parker HG, Ostrander EA (2016). Whole-genome sequence, SNP chips and pedigree structure: building demographic profiles in domestic dog breeds to optimize genetic-trait mapping. Dis. Model. Mech..

[55] Ouborg NJ, Pertoldi C, Loeschcke V, Bijlsma RK, Hedrick PW, Pertoldi C (2010). Conservation genetics in transition to conservation genomics. Trends Genet..

[56] Boscari E, Barmintseva A, Pujolar JM, Doukakis P, Mugue N, Congiu L (2014). Species and hybrid identification of sturgeon caviar: A new molecular approach to detect illegal trade. Mol. Ecol. Resour..

[57] Pilot M, Greco C, vonHoldt BM, Jędrzejewska B, Randi E, Jędrzejewski W (2014). Genome-wide signatures of population bottlenecks and diversifying selection in European wolves. Heredity..

[58] Schoenebeck JJ, Hutchinson SA, Byers A, Beale HC, Carrington B, Faden DL (2012). Variation of BMP3 Contributes to Dog Breed Skull Diversity. PLoS Genet..

[59] Mastorakos G, Ilias I (2003). Maternal and fetal hypothalamic-pituitary-adrenal axes during pregnancy and postpartum. Ann. N. Y. Acad. Sci..

[60] Arnott ER, Peek L, Early JB, Pan AYH, Haase B, Chew T (2015). Strong selection for behavioural resilience in Australian stock working dogs identified by selective sweep analysis. Canine Genet. Epidemiol..

[61] Parker J, Tsagkogeorga G, Cotton JA, Liu Y, Provero P, Stupka E (2013). Genome-wide signatures of convergent evolution in echolocating mammals. Nature.

[62] Le Guédard S, Faugère V, Malcolm S, Claustres M, Roux A-F (2007). Large genomic rearrangements within the PCDH15 gene are a significant cause of USH1F syndrome. Mol. Vis..

[63] Schweizer RM, VonHoldt BM, Harrigan R, Knowles JC, Musiani M, Coltman D (2016). Genetic subdivision and candidate genes under selection in North American grey wolves. Mol. Ecol..

[64] Miyadera K, Acland GM, Aguirre GD (2012). Genetic and phenotypic variations of inherited retinal diseases in dogs: The power of within- and across-breed studies. Mamm. Genome..

[65] Li Y, Von Holdt BM, Reynolds A, Boyko AR, Wayne RK, Wu DD (2013). Artificial selection on brain-expressed genes during the domestication of dog. Mol. Biol. Evol..

[66] Pfahler S, Distl O (2015). Effective population size, extended linkage disequilibrium and signatures of selection in the rare dog breed Lundehund. PLoS One.

[67] Ostrander EA (2012). Both ends of the leash–the human links to good dogs with bad genes. N. Engl. J. Med..

[68] Freedman AH, Schweizer RM, Ortega-Del Vecchyo D, Han E, Davis BW, Gronau I (2016). Demographically-Based Evaluation of Genomic Regions under Selection in Domestic Dogs. PLoS Genet..

[69] Moroudi RS, Masoudi AA, Vaez Torshizi R, Zandi M (2014). Identification of learning and memory genes in canine; promoter investigation and determining the selective pressure. Mol. Biol. Rep..

[70] Persson ME, Wright D, Roth LSV, Batakis P, Jensen P (2016). Genomic regions associated with interspecies communication in dogs contain genes related to human social disorders. Sci. Rep..

[71] Albert FW, Carlborg Ö, Plyusnina I, Besnier F, Hedwig D, Lautenschläger S (2009). Genetic architecture of tameness in a rat model of animal domestication. Genetics..

[72] Bélteky J, Agnvall B, Jensen P (2017). Gene expression of behaviorally relevant genes in the cerebral hemisphere changes after selection for tameness in Red Junglefowl. PLoS One..

[73] Wilkins AS, Wrangham RW, Tecumseh Fitch W (2014). The “domestication syndrome” in mammals: A unified explanation based on neural crest cell behavior and genetics. Genetics..

[74] Benazzo A, Trucchi E, Cahill JA, Maisano Delser P, Mona S, Fumagalli M (2017). Survival and divergence in a small group: The extraordinary genomic history of the endangered Apennine brown bear stragglers. Proc. Natl. Acad. Sci..

[75] Chase K, Jones P, Martin A, Ostrander EA, Lark KG (2009). Genetic mapping of fixed phenotypes: Disease frequency as a breed characteristic. J. Hered..

[76] Boyko AR (2011). The domestic dog: man’s best friend in the genomic era. Genome Biol..

[77] Yang H, Wang G, Wang M, Ma Y, Yin T, Fan R, et al. The origin of chow chows in the light of the East Asian breeds. BMC Genomics. 2017;18:174. 10.1186/s12864-017-3525-9 PMC5312535 28201986

[78] Cattanach BM, Dukes-McEwan J, Wotton PR, Stephenson HM, Hamilton RM (2015). A pedigree-based genetic appraisal of Boxer ARVC and the role of the Striatin mutation. Vet. Rec..

[79] Downs LM, Wallin-Håkansson B, Boursnell M, Marklund S, Hedhammar Å, Truvé K, et al. A frameshift mutation in Golden Retriever dogs with progressive retinal atrophy endorses SLC4A3 as a candidate gene for human retinal degenerations. PLoS One. 2011;6:e21452. 10.1371/journal.pone.0021452 PMC3124514 21738669

[80] Parker HG, Kukekova AV, Akey DT, Goldstein O, Kirkness EF, Baysac KC (2007). Breed relationships facilitate fine-mapping studies: a 7.8-kb deletion cosegregates with Collie eye anomaly across multiple dog breeds. Genome Res.

[81] van Rooy D, Arnott ER, Early JB, McGreevy P, Wade CM (2014). Holding back the genes: limitations of research into canine behavioural genetics. Canine Genet. Epidemiol.

