# Supplementary figures and images for: Wolf outside, dog inside? The genomic make-up of the Czechoslovakian Wolfdog
Source: BMC Genomics. 2018 Jul 13;19:533. doi: 10.1186/s12864-018-4916-2 (PMC6043967; doi:10.1186/s12864-018-4916-2)

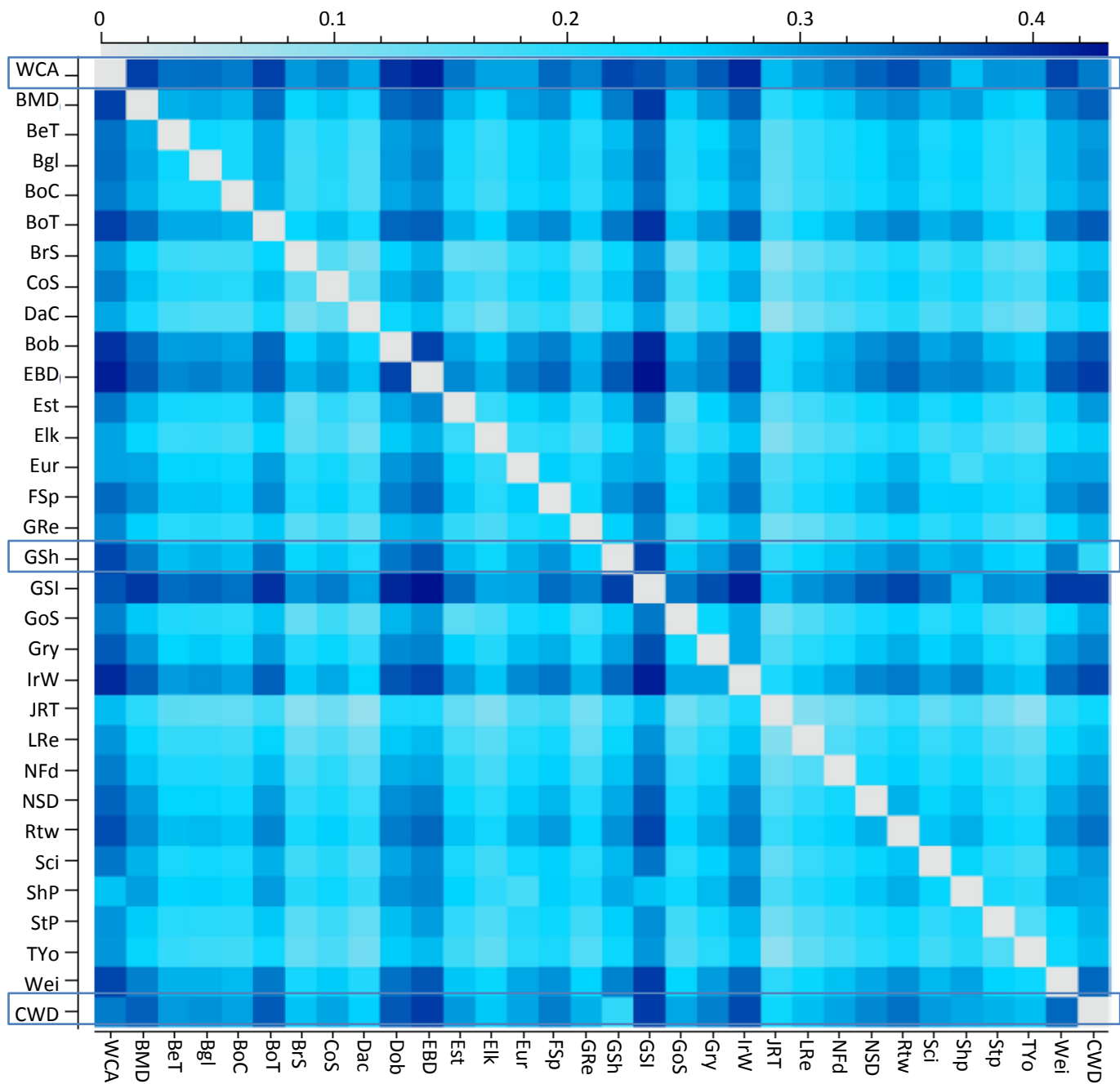

Supplement: Supplementary file 1 — Figure S1. FST heat plot matrix of the genetic distances among groups computed from the 126k dataset in SVS. The most distant breed to Carpathian wolves (WCA) is the English Bulldog (EBD) while the closest one is the ancient breed Shar-Pei (ShP). As expected the least differentiated breed from the Czechoslovakian Wolfdog (CWD) is the German Shepherd (GSh). (PDF 200 kb) [file 12864_2018_4916_MOESM1_ESM.pdf]

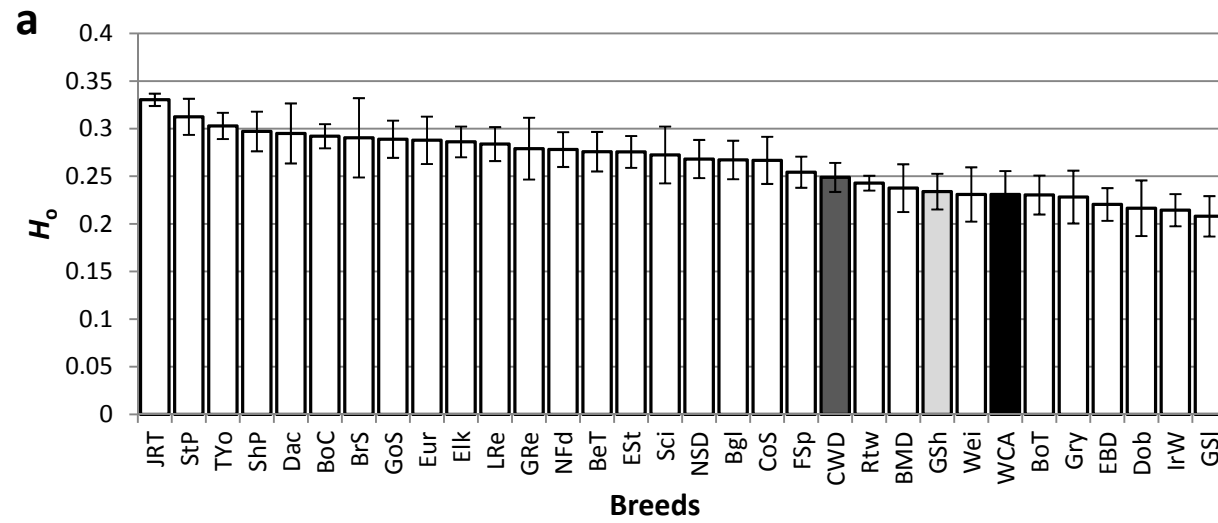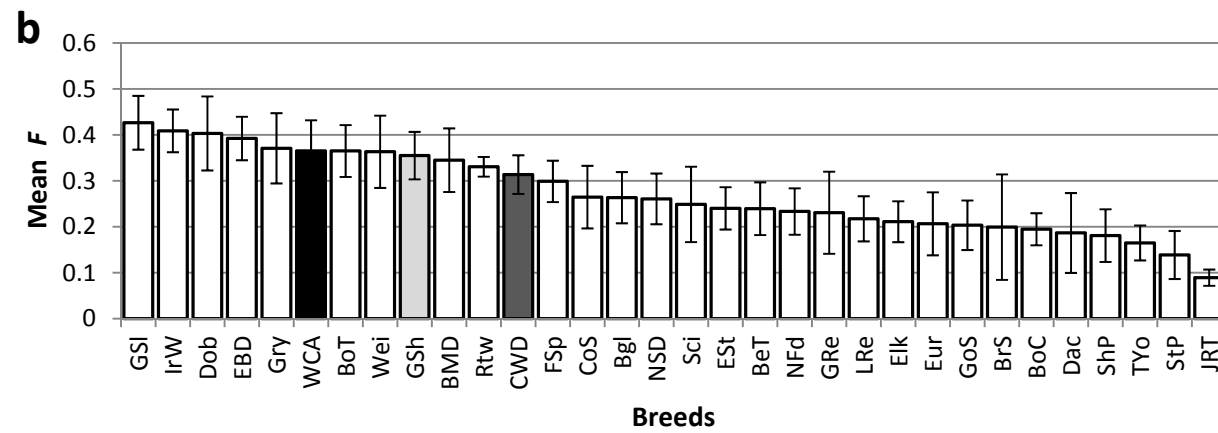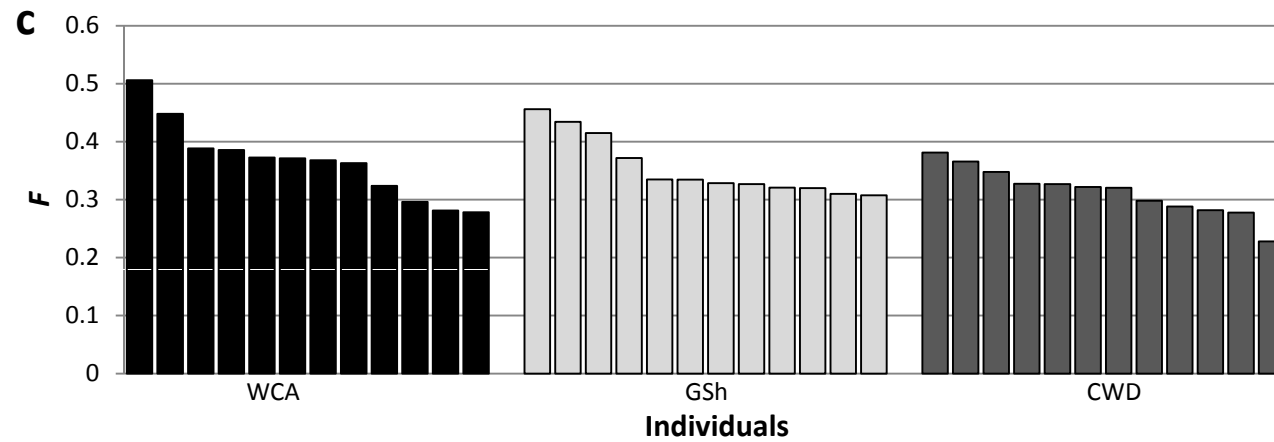

Supplement: Supplementary file 2 — Figure S2. Genetic variability indexes computed in SVS using the 126k SNP dataset. a Mean values of observed heterozygosity (Ho) within groups. Czechoslovakian Wolfdogs (in dark gray) show higher levels of heterozygosity than parental populations (Carpathian wolves in black and German Shepherds in light grey), as expected from the recent crossings that originated the breed, but lower than most breeds. Bars indicate standard deviations. b Plots of the mean inbreeding coefficient F per breed. Czechoslovakian Wolfdogs show a mean F value intermediate among the other breeds but lower than both parental populations. c: from left to right: individual F values for Carpathian wolf (black histograms), German Shepherd (light grey histograms) and Czechoslovakian Wolfdog (dark gray histograms) groups. Bars indicate standard deviations. (PDF 94 kb) [file 12864_2018_4916_MOESM2_ESM.pdf]

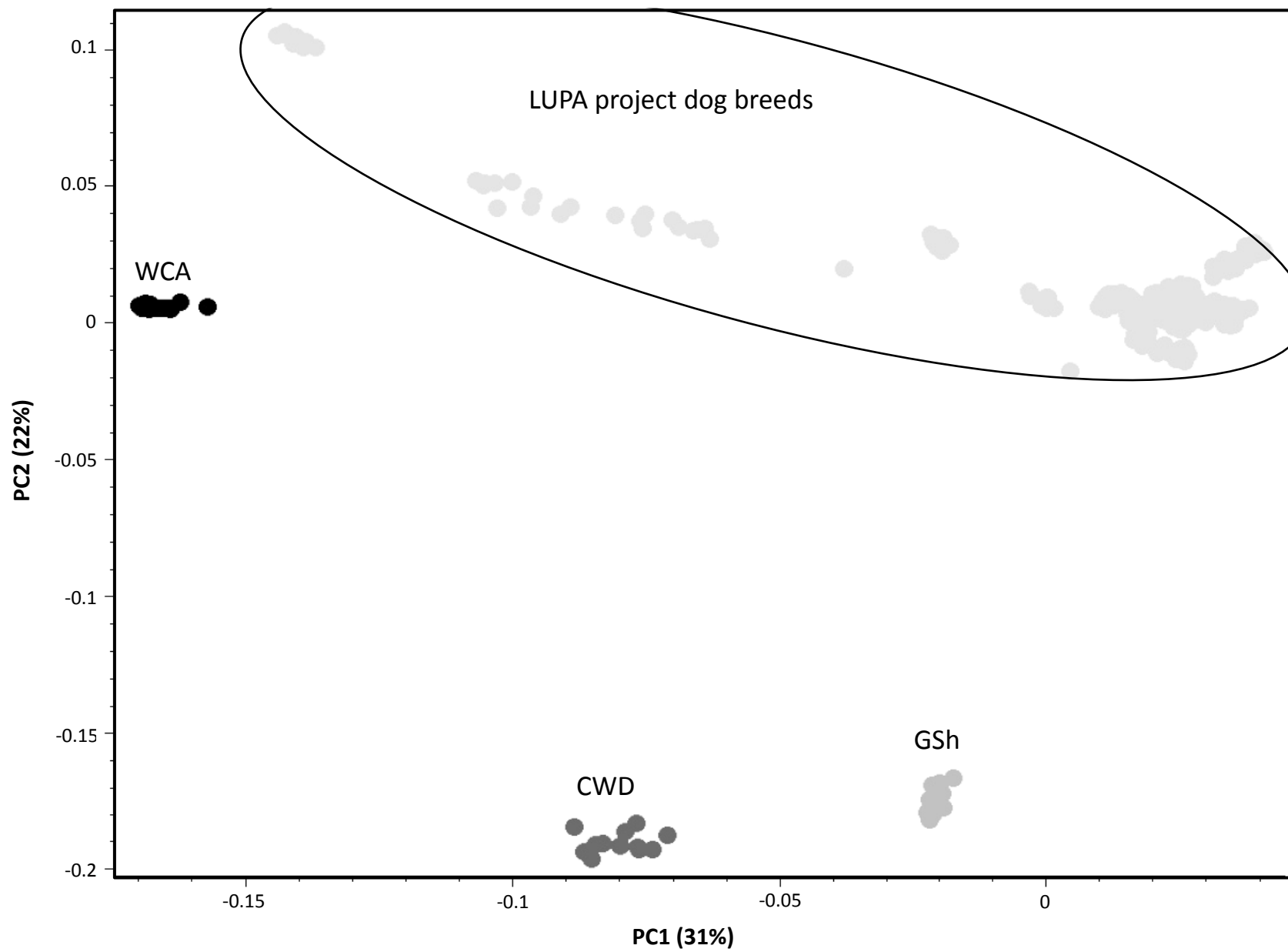

Supplement: Supplementary file 3 — Figure S3. PC1 vs. PC2 results from an exploratory principal component analysis (PCA) computed in SVS on the 126k SNP dataset and including dogs from 30 pure breeds (extrapolated from the available LUPA project dataset; top side of the graph, in grey inside the circle), Carpathian wolves (WCA; black dots to the left), German Shepherds (GSh; light grey dots in the bottom), and Czechoslovakian Wolfdogs (CWD; dark gray dots in the bottom). The two axes are not to scale, in order to better distinguish individuals along PC2. (PDF 202 kb) [file 12864_2018_4916_MOESM3_ESM.pdf]

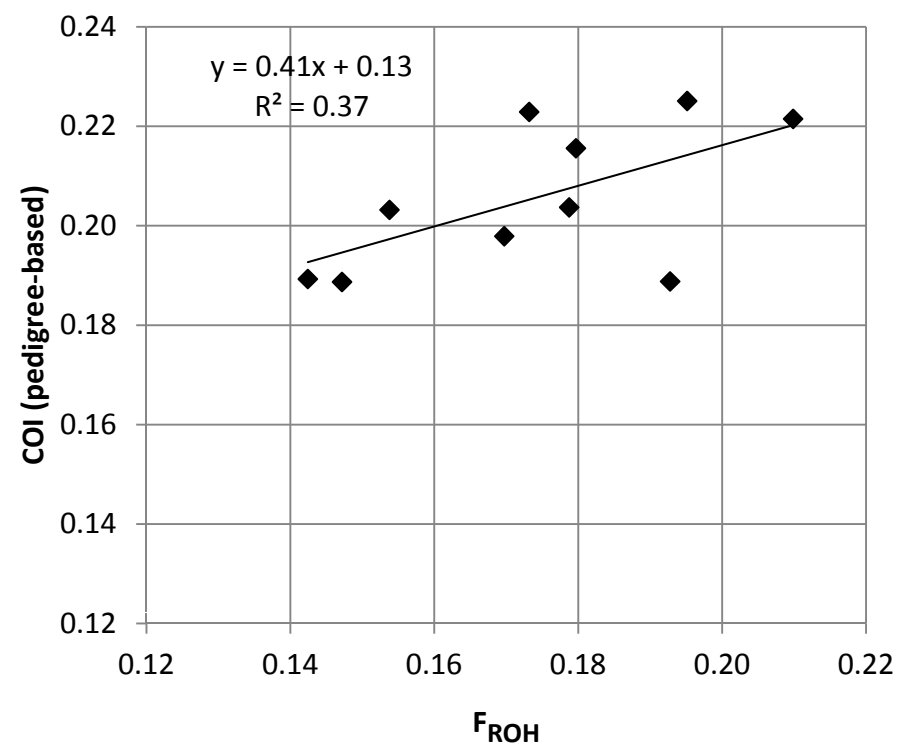

Supplement: Supplementary file 4 — Figure S4. Comparison between the individual frequency of ROHs (FROH), calculated in SVS as the proportion of ROHs on the genome length spanned by the analysed SNPs (on the horizontal axis), and the individual Wright’s inbreeding coefficient (COI), estimated from the pedigrees with the software U-WGI (on the vertical axis). The two inbreeding indexes are significantly (p < 0.01) correlated. (PDF 71 kb) [file 12864_2018_4916_MOESM4_ESM.pdf]

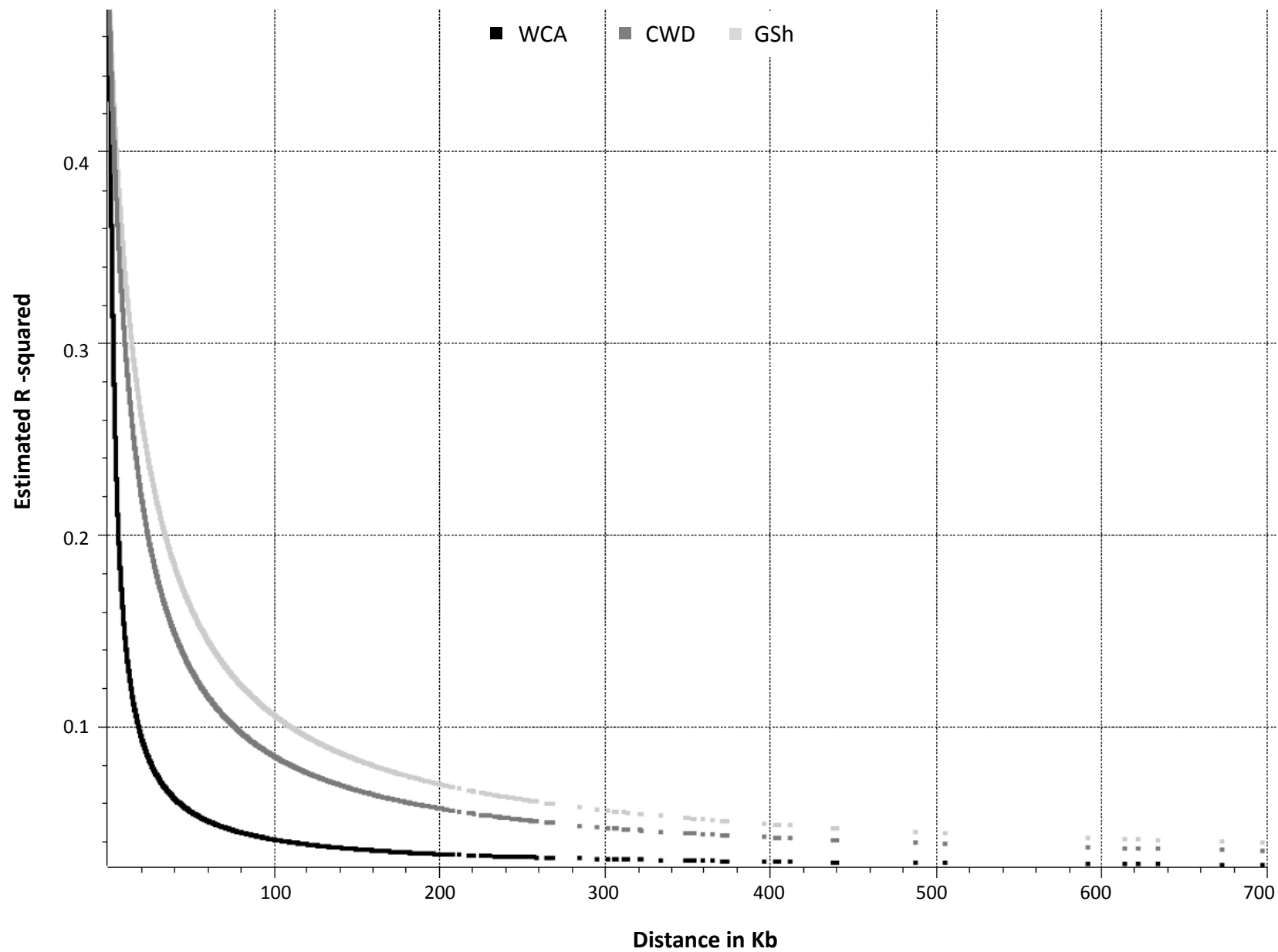

Supplement: Supplementary file 5 — Figure S5. Linkage disequilibrium (LD) decay plot. The vertical axis indicates the mean Estimated R-squared (r2), and the horizontal axis indicates the distance in kb at which LD decays. (PDF 220 kb) [file 12864_2018_4916_MOESM5_ESM.pdf]

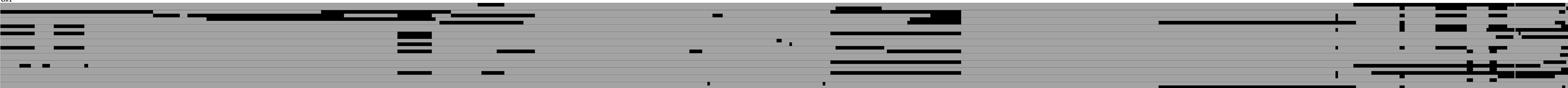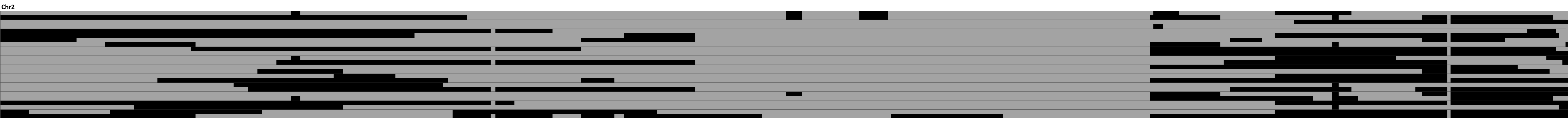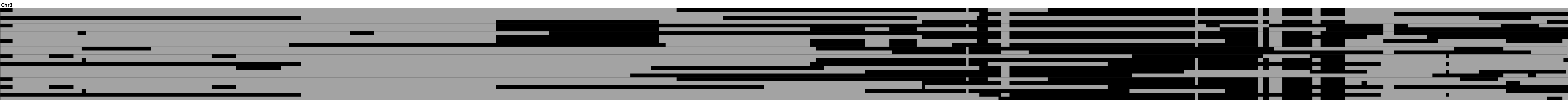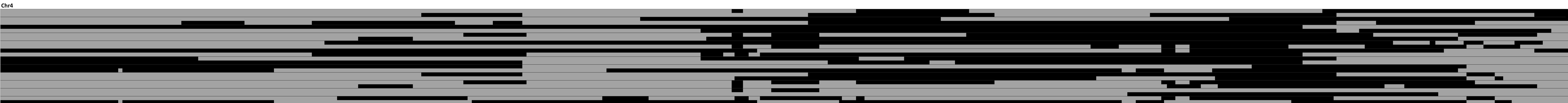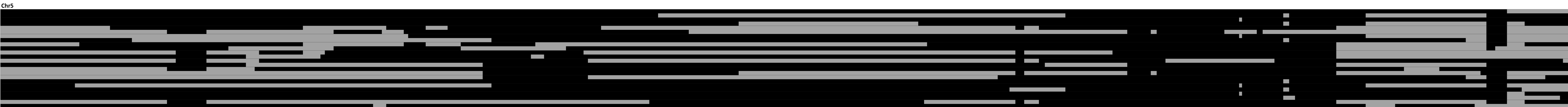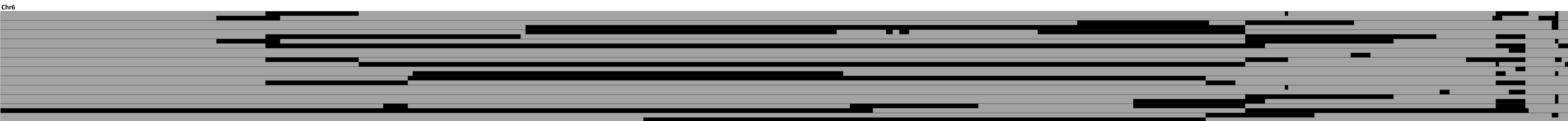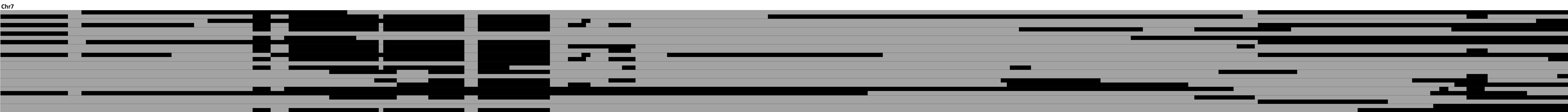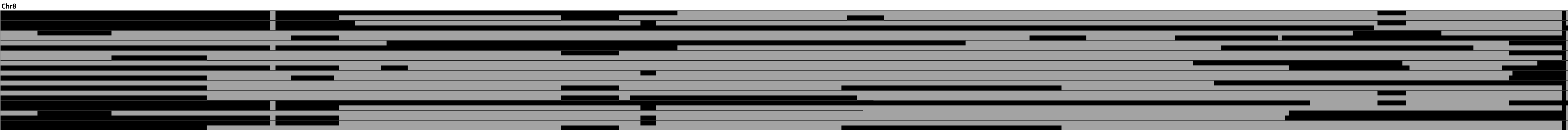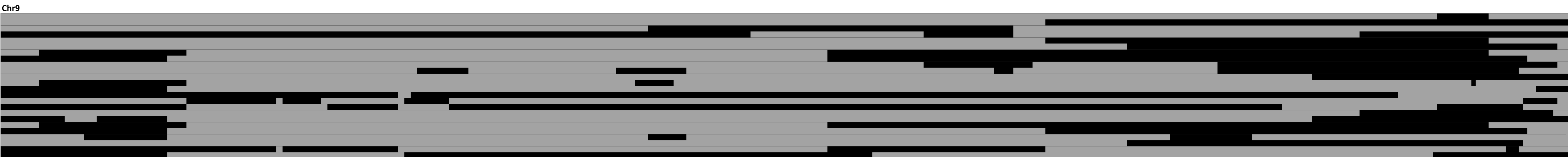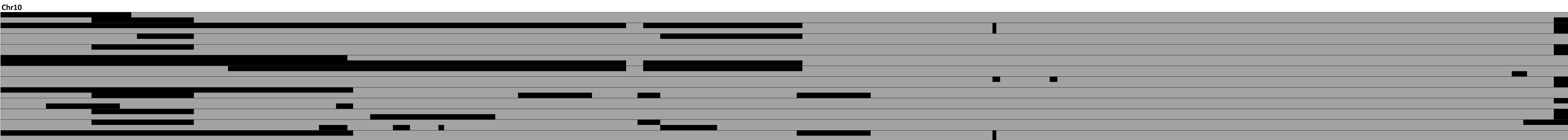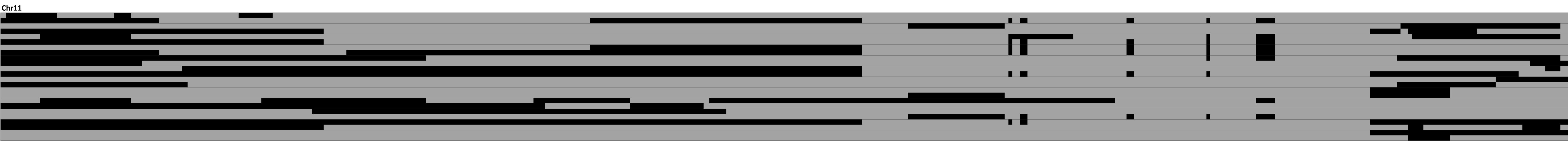

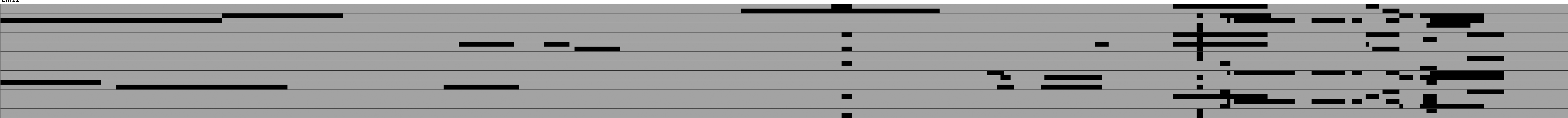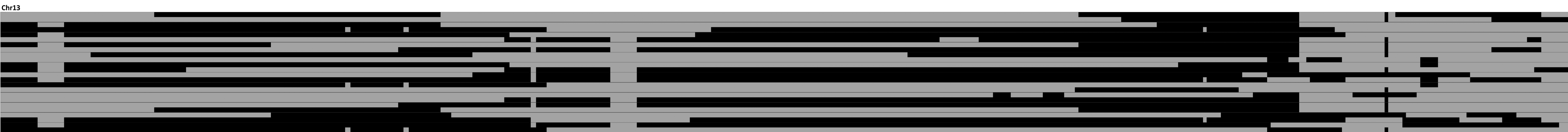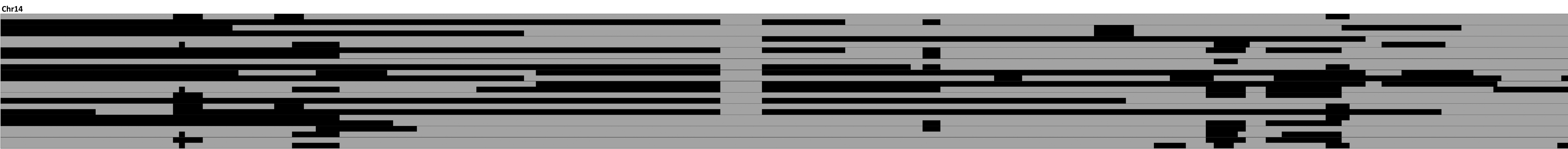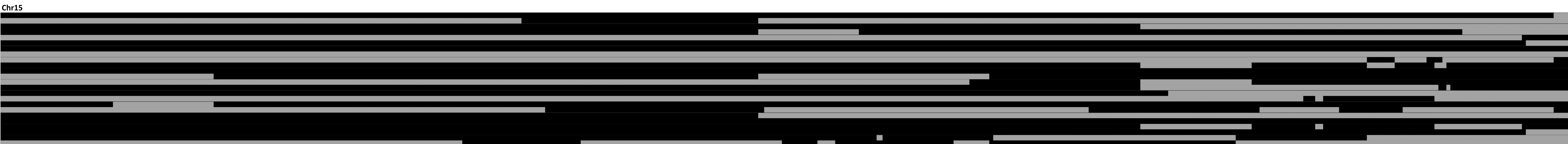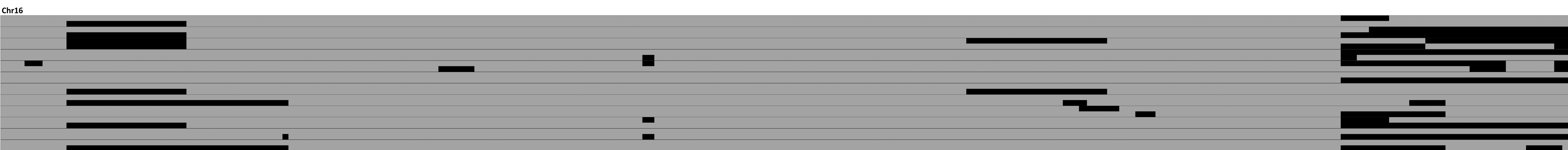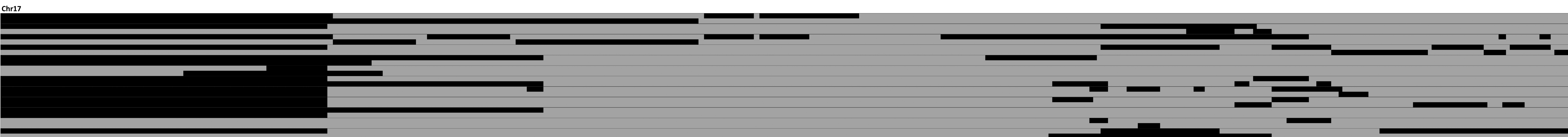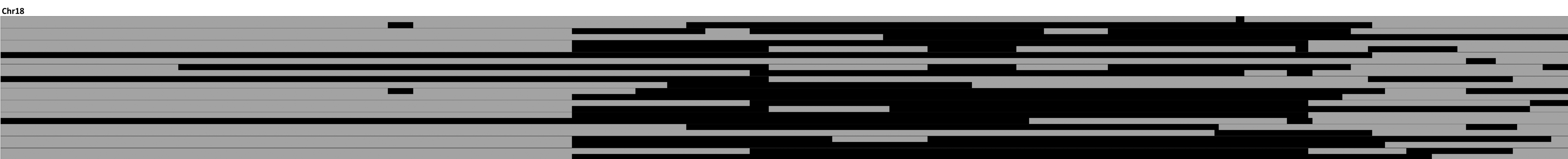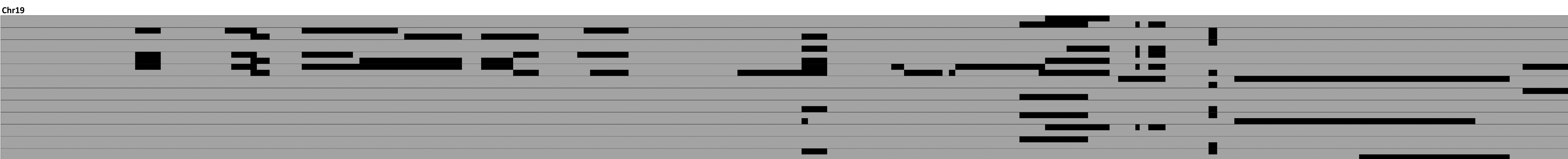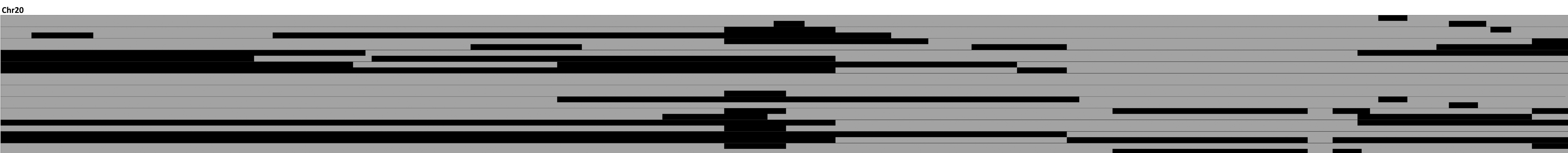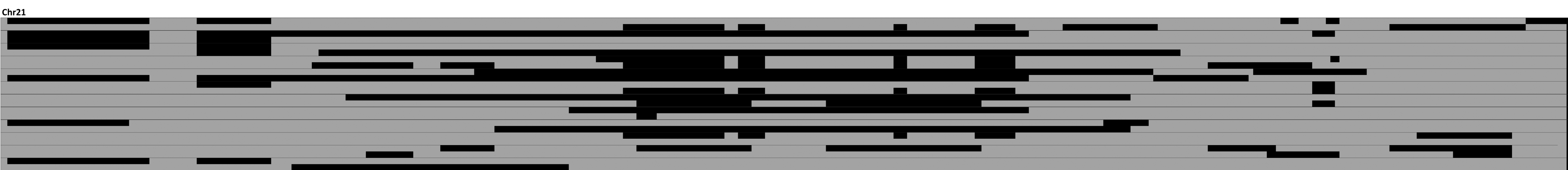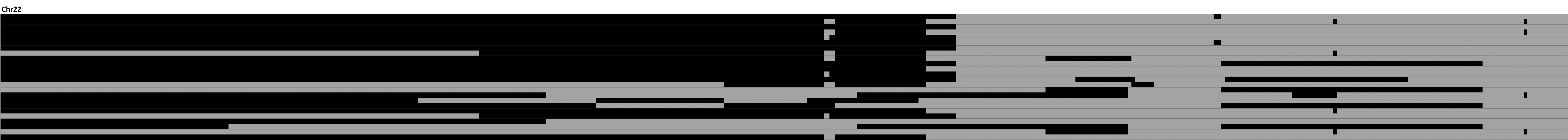

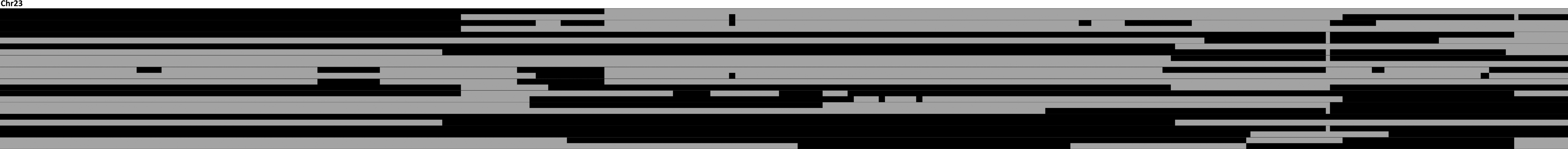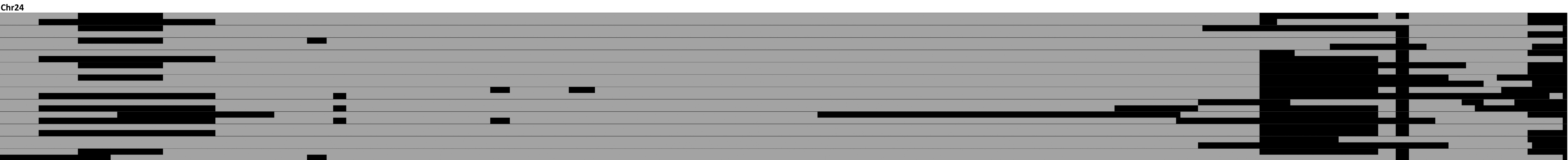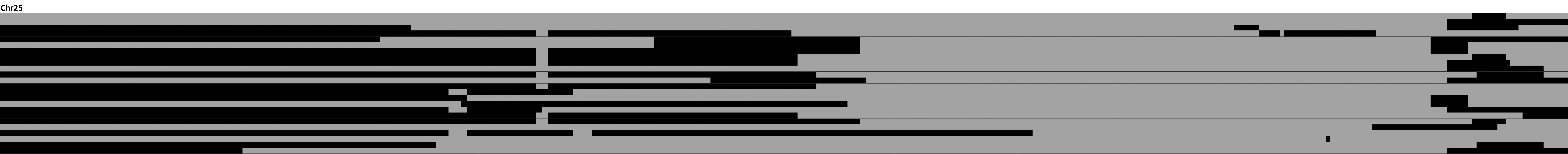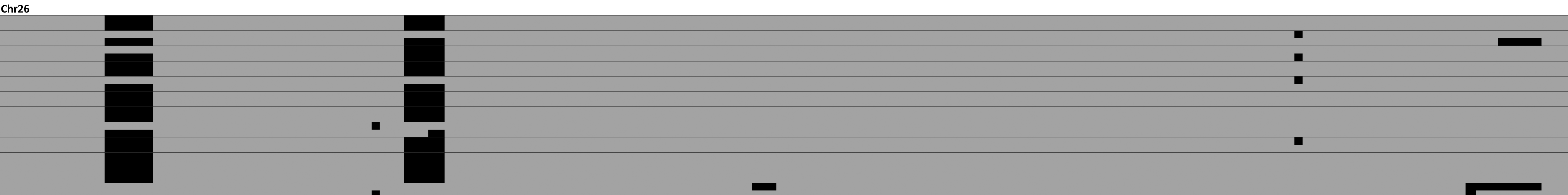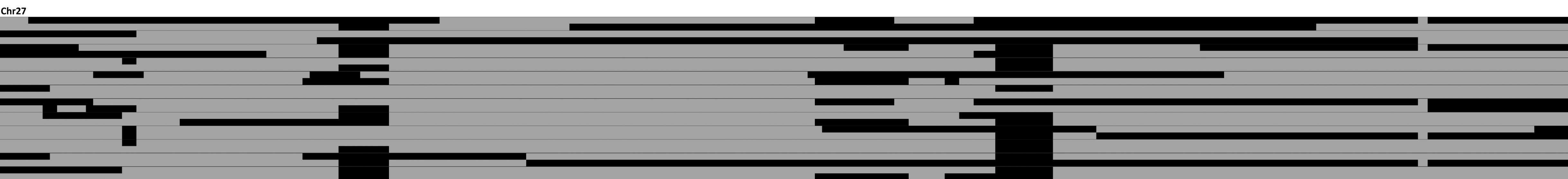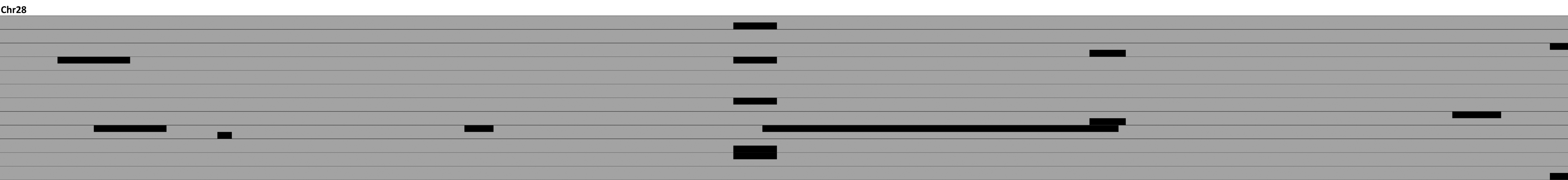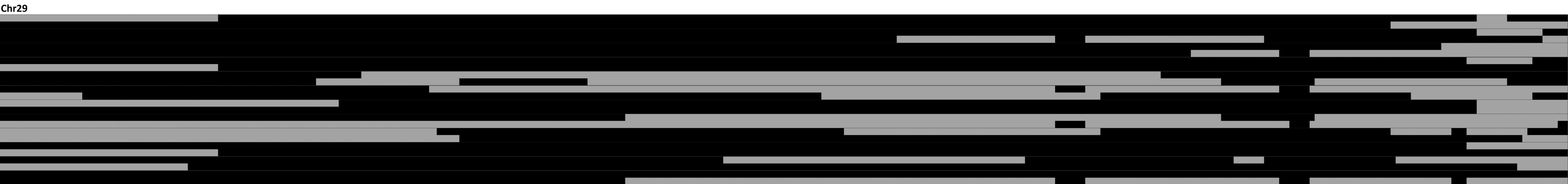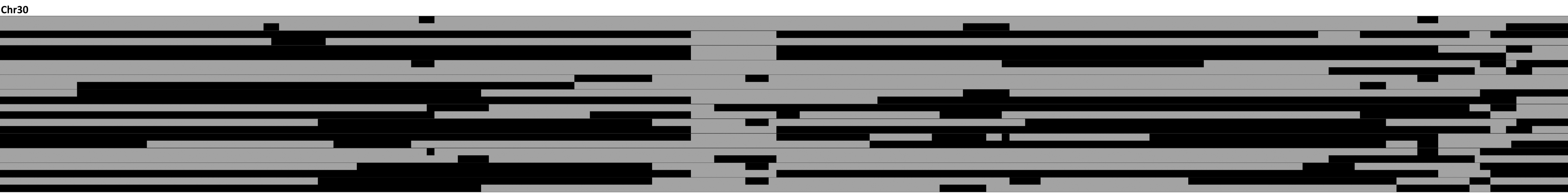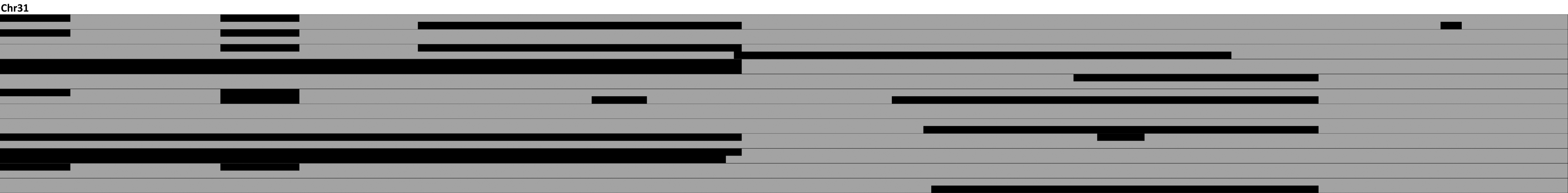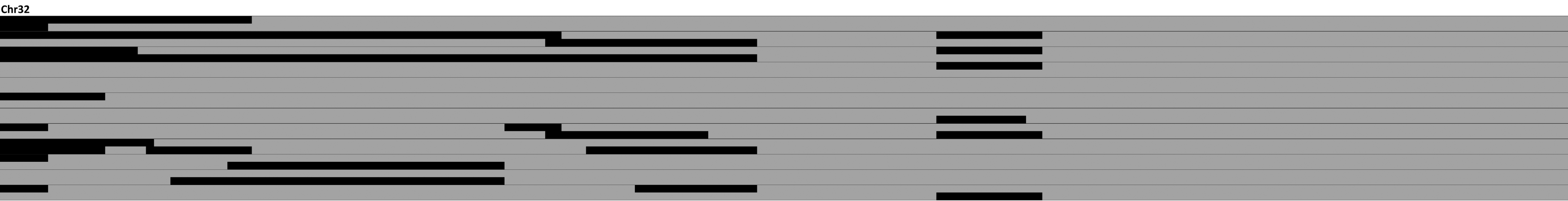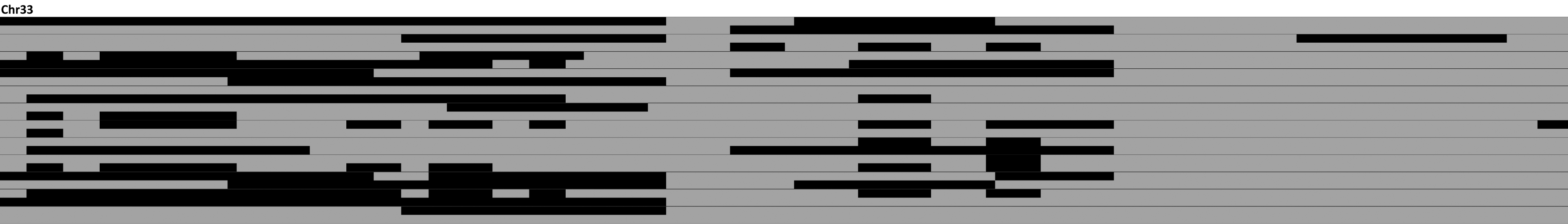

Chr34

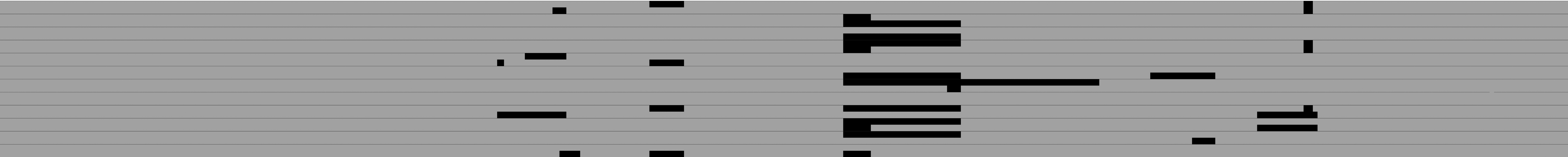

Chr35

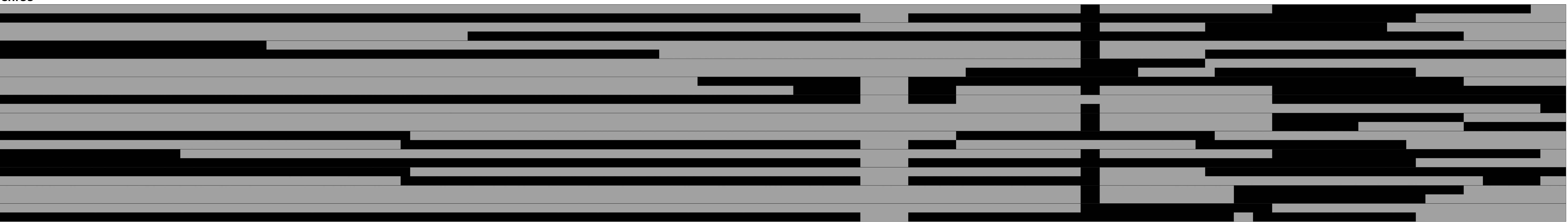

Chr36

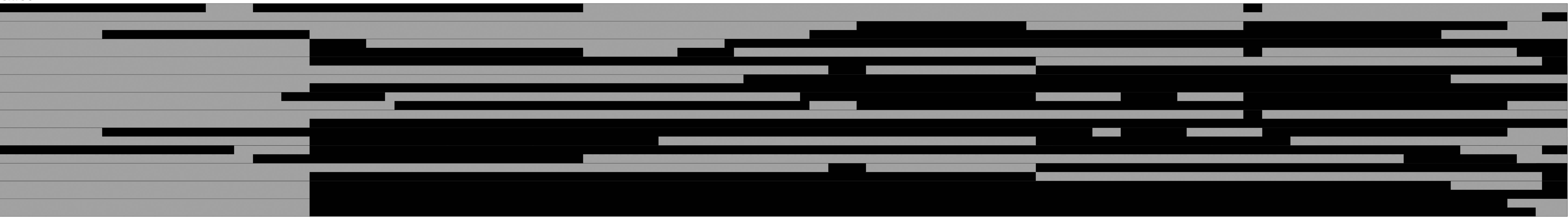

Chr37

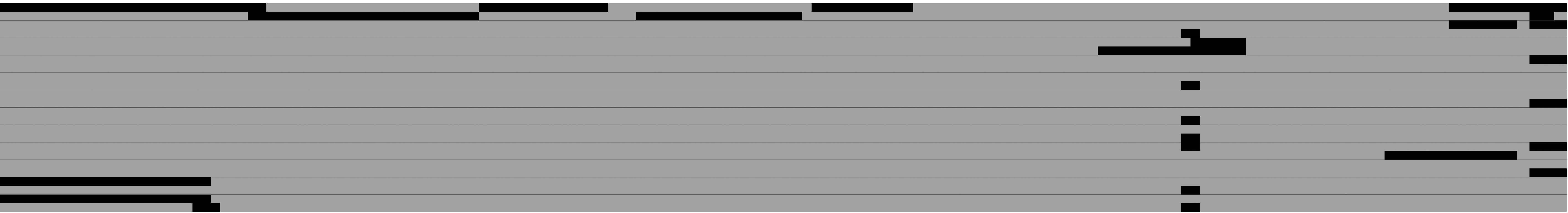

Chr38

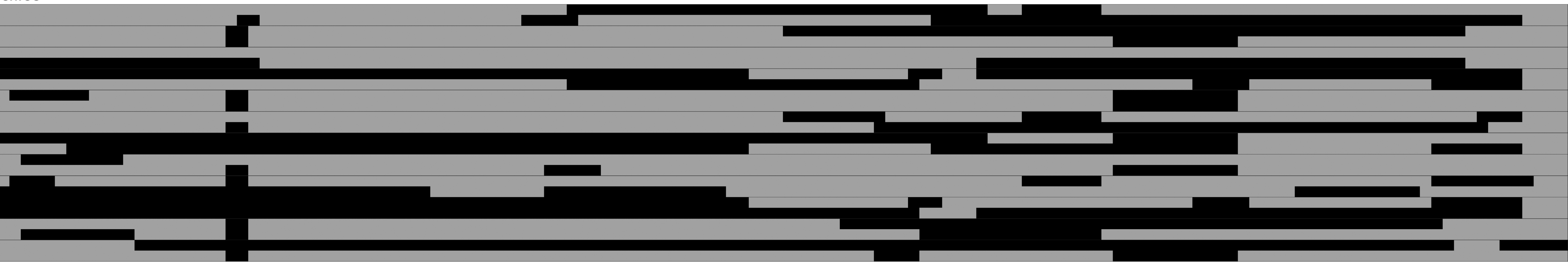

Supplement: Supplementary file 6 — Figure S6. Graphical representation, for each chromosome of each analysed Czechoslovakian Wolfdog, of the ancestry components identified by PCAdmix based on the analysis of 10-SNP haplotype blocks. Each horizontal bar represents the two homologous chromosomes of an individual showing in black the genomic regions assigned as wolf-like and in light grey those assigned as dog-like. (PDF 10810 kb) [file 12864_2018_4916_MOESM6_ESM.pdf]
