# Supplementary material for: Wolf outside, dog inside? The genomic make-up of the Czechoslovakian Wolfdog
Source: BMC Genomics. 2018 Jul 13;19:533. doi: 10.1186/s12864-018-4916-2 (PMC6043967; doi:10.1186/s12864-018-4916-2)
Supplement: Supplementary file 8 — Figure S7a. BGC alpha parameter outlier SNPs. Values lower than 0 indicate excess of wolf alleles, values higher than 0 indicate excess of dog alleles. BGC significant outliers are indicated by blue crosses (top or bottom 1% of the empirical distribution of values) and by red dots (95% credibility intervals of 10,000 iterations not including 0). Figure S7b. BayeScan outlier SNPs detected comparing differences in allele frequency between Czechoslovakian Wolfdogs and German Shepherds (right) and between Czechoslovakian Wolfdogs and Carpathian wolves (left). The vertical axis indicates mean FST values between populations, and the horizontal axis indicates the logarithm of posterior odds (log(PO)). The vertical line indicates the log(PO) value corresponding to the false discovery rate threshold of 0.05. Loci on the right of this line are putatively under selection. (PDF 312 kb) [file 12864_2018_4916_MOESM8_ESM.pdf]

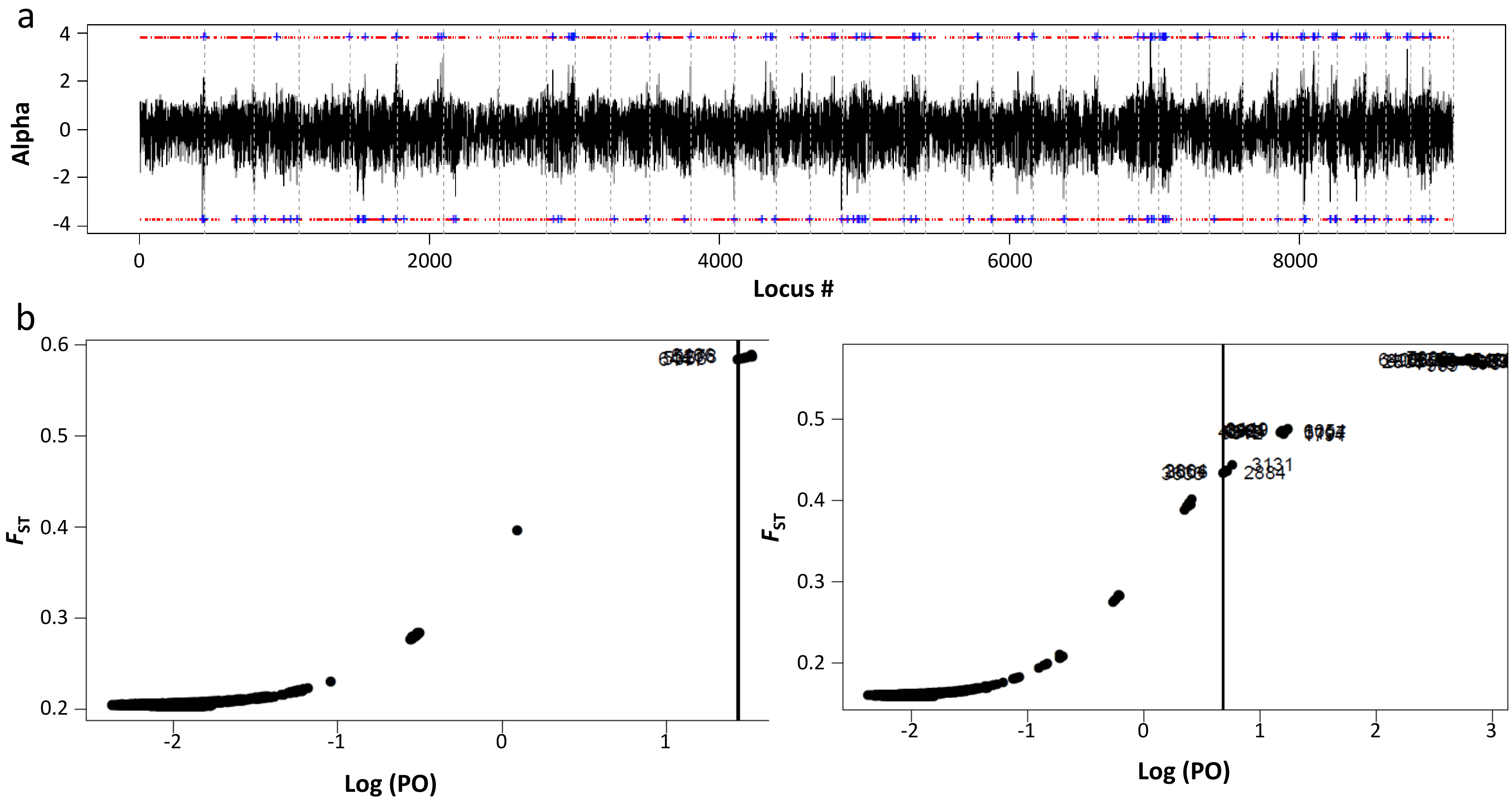
a
Alpha
4
2
0
-2
-4
0
2000
4000
6000
8000
Locus #
b
0.6
0.5
0.4
0.3
0.2
F_{ST}
-2
-1
0
1
Log (PO)
0.5
0.4
0.3
0.2
F_{ST}
3131
2884
-2
-1
0
1
2
3
Log (PO)
